# Poly(A)+ selection limits detection of long and alternatively spliced transcripts compared with rRNA depletion in RNA-Sequencing

**DOI:** 10.1186/s12864-026-12944-z

**Published:** 2026-05-13

**Authors:** Swethaa Natraj Gayathri, Victoria Lillback, Bjarne Udd, Peter Hackman, Marco Savarese, Ali Oghabian

**Affiliations:** 1 University of Helsinki, Helsinki, Finland; 2 Folkhälsan Research Center, Helsinki, Finland

**Keywords:** RNA-Sequencing, rRNA, Poly(A)+, Transcriptomics, TTN, Muscle

## Abstract

The eukaryotic transcriptome diversity arises largely from alternative splicing. One of the widely used high-throughput methods to study this diversity is RNA sequencing. RNA sequencing has become a cornerstone of both basic biology and precision medicine, facilitating the quantification of gene and transcript expression, as well as the characterization of alternative splicing events and regulatory biological pathways in these studies. As there is a wide interest in studying non-ribosomal RNAs, which constitute about 20% of cellular RNAs, it is common to either select for poly(A)+ RNAs or to deplete ribosomal RNAs during the library preparation stage of RNA sequencing. At the time of library preparation, poly(A)+ selected RNA-Seq captures the polyadenylated transcripts, whereas rRNA-depleted RNA-Seq pools a broader spectrum of RNA species, including non-polyadenylated and premature transcripts. Using blood and skeletal muscle transcriptomics datasets, we examined how these two library enrichment techniques influence transcript representation, transcript-body coverage, and splice junction detection. We observed that poly(A)+ selected libraries display length-dependent differences, reduced splice junction representation and pronounced 3’ end coverage bias for transcripts of total transcription length over 5 kb. In contrast, rRNA depletion provides a more uniform 5′-3′ coverage, an improved detection of splice junctions, and a robust detection of long disease-relevant transcripts. These differences are evident in the detection of extremely large transcripts, such as the sarcomeric genes *OBSCN* (~ 39 kb) and *TTN* (> 100 kb). This study discusses how RNA-Seq library preparation techniques capture different RNA types and emphasizes the importance of interpreting poly(A)+ selected and rRNA depleted data in the appropriate biological and clinical contexts.

**Supplementary Information:**

The online version contains supplementary material available at 10.1186/s12864-026-12944-z.

## Background

RNA sequencing or RNA-Seq enables the characterization of gene expression patterns, alternative splicing, and regulatory pathways in different samples and conditions. It is widely used in a broad range of research fields, including life sciences, clinical diagnostics, and in the development of novel therapeutics [1–3]. As ribosomal RNAs (rRNAs) account for more than 80% of the total RNA in the eukaryotic cells [4, 5], often during library preparation either poly(A)+ selection is performed, to enrich for polyadenylated mRNA, or ribosomal RNA (rRNA) depletion is applied to remove rRNA and retain the remaining RNA population. These two approaches have previously been benchmarked and compared for the composition of the detected RNAs, quantification of gene expression, and ability to detect lowly expressed genes [6–8]. In particular, rRNA depleted RNA-Seq has been reported to be capable of detecting more long non-coding RNAs (lncRNAs) and to better detect the lowly expressed transcripts [6–9]. However, this approach also retains immature RNA, including degraded transcripts, which may complicate data interpretation [6]. A study by Kapranov et al. introduced the concept of ‘dark matter’ RNA [10]. They reported that extensive population of non-ribosomal and non-mitochondrial transcripts, including unannotated intergenic RNAs, that remain invisible and underrepresented in poly(A)+ based approaches, are detected by rRNA-depleted RNA-Seq [10]. Moreover, the sequence reads achieved from rRNA depleted RNA-Seq cover the gene body (i.e. from 5’ to 3’ end of the gene) more uniformly [8], whereas poly(A)+ selection exhibits a bias toward detecting the 3’ ends of these transcripts [11]. These findings reveal that both, the RNA-Seq results we observe and our biological interpretation of the data, are strongly influenced by the choice of library preparation method. However, one critical aspect that remains insufficiently explored is the extent to which different RNA-Seq library enrichment methods affect the detection of transcripts of varying sizes. This is important since long transcripts with complex genetic architecture, diverse splicing patterns and critical biological functions present challenges that can complicate their detection by RNA-Seq [12–14]. Furthermore, the size of mRNAs encoded by genes in human genome can exceed 50 kb, which is too large to be fully detectable by short-read and even some existing long-read RNA-Seq platforms (e.g. Iso-Seq by PacBio) [15, 16]. These long-isoform-coding genes are associated with diverse functions including neuronal processes, embryonic development and ageing [14, 17, 18]. Three of the genes with the longest mRNAs in humans, namely *TTN*, *NEB* and *OBSCN*, encode for sarcomeric proteins that are essential in muscle formation and function [19–22]. Therefore, we propose that the limited detection sensitivity and non-uniform coverage of long mRNAs in poly(A)+ RNA-Seq data particularly impact research areas such as ageing, neuronal development, muscle biology, and disorders affecting neuronal and muscle tissues, although similar biases are expected across all biological research fields. Importantly, this challenge extends to the clinical diagnostic setting, where RNA-Seq is increasingly used to interpret the pathogenicity of genomic variants, especially splice variants in disease-associated genes [23–25]. A critical step in the clinical interpretation of RNA-Seq data involves visualizing candidate disease-causing variants in the Integrative Genomics Viewer (IGV) [26], which is instrumental in identifying splice variants that reveal aberrant splicing patterns. To detect the potential disease-associated RNA splicing and gene expression dysregulation, several computational tools, including DROP [27], FRASER [28], and OUTRIDER [29] have been developed. These tools enable detection of aberrant splicing and transcriptional outliers by integrating statistical models and multi-omics data, thereby increasing sensitivity and diagnostic yield for rare disease-associated variants.

Here, we aim to systematically compare poly(A)+ selection and rRNA depletion, two commonly used RNA-Seq library enrichment methods, by analyzing data from varied human tissue types (i.e. blood and skeletal muscle). We study transcript-body coverage, gene expression detection, and splice junction detection across transcripts of different transcription length groups, focusing on aspects that have not been thoroughly explored previously. We further illustrate how these differences can influence the interpretation of disease-associated variants in very large transcripts like *TTN* (> 100 kb).

## Results

### Ribodepletion RNA-Seq reads offer more uniform transcript coverage

**Fig. 1:**
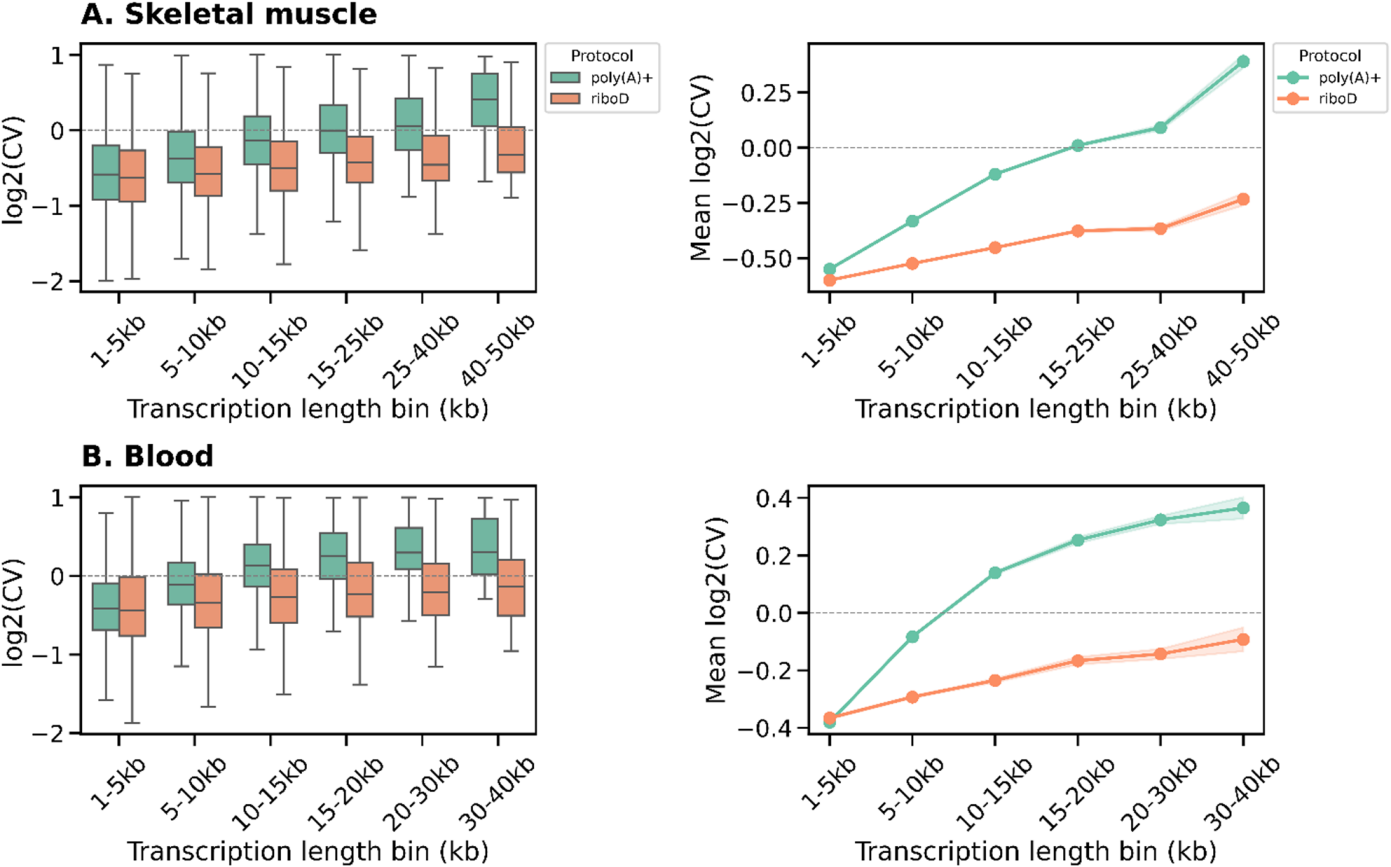
Transcript coverage variation in RNA-Seq. Boxplot (left) show coefficient of variation of read coverage (CV) across transcripts grouped by transcription length (TL). Each box indicates the interquartile range; whiskers represent the spread of values across genes, not error bars. Line plot (right) highlights the mean trend from the boxplot. **A** Skeletal muscle RNA-Seq indicates for transcription length > 5 kb, riboD (rRNA depletion) libraries consistently show lower variation of read coverage than poly(A)+ enrichment. **B** Blood RNA-Seq also indicate that for transcripts > 5 kb, rRNA depletion consistently shows lower variation than poly(A)+ enrichment

We compared the distribution of mapped sequence reads across the transcript body of different transcription lengths when using poly(A)+ selection versus rRNA depletion for library enrichment. For this analysis, twenty-three skeletal muscle (SM) samples were run in rRNA depleted RNA-Seq and a different set of twenty-three samples were run in poly(A)+ enriched RNA-Seq. To assess read coverage uniformity, the coefficient of variation (CV) of read coverage across transcripts of each gene was plotted against transcription length (TL). (Fig. 1A). rRNA depleted RNA-Seq consistently showed lower CV values compared to those of poly(A)+ selected libraries, particularly for transcripts longer than 5 kb, indicating more uniform transcript coverage when using rRNA depletion for skeletal muscle RNA-Seq cohorts (Supplementary 1). When examining the TL measurements for transcripts with transcript per million (TPM) > 1, poly(A)+ selected RNA-Seq detected only a limited number of long transcripts (> 40 kb): three transcripts in the 40–50 kb range (*CWC27*,* FTX*,* MYO5A*), one between 50 and 100 kb (*CCDC26*), and one above 100 kb (*TTN*) (Fig. 1A). In contrast, the rRNA depleted RNA-Seq detected a higher number of long transcripts (Fig. 1A): six of which constitute in the 40–50 kb range (*ARID1B*,* DST*,* KIAA1109*,* MAPK10*,* MYO5A*,* NF1*), four are in the 50–100 kb range (*ANK2*,*CCDC26*,* KMT2C*,* MACF1*), and one over 100 kb (*TTN*).

We also checked CV distribution against TL in blood samples (values for all annotated genes in Gencode v39 in Supplementary 1). In blood RNA-Seq, only a few long transcripts with TPM >1 (> 40 kb) were detected. Interestingly, only rRNA depleted RNA-Seq could detect transcripts larger than 50 kb, namely *ANK2* (average TPM 6.2) and *TTN* (average TPM 2.8), as well as long non-coding transcripts *CCDC26*,* KCNQ1OT1*,* HELLPAR*. Within the 1–40 kb range transcripts, rRNA depleted RNA-Seq consistently showed lower CV than poly(A)+ libraries (Fig. 1B). This also highlights the reduced sensitivity of poly(A)+ selection for large genes in blood (Fig. 1B).

**Fig. 2:**
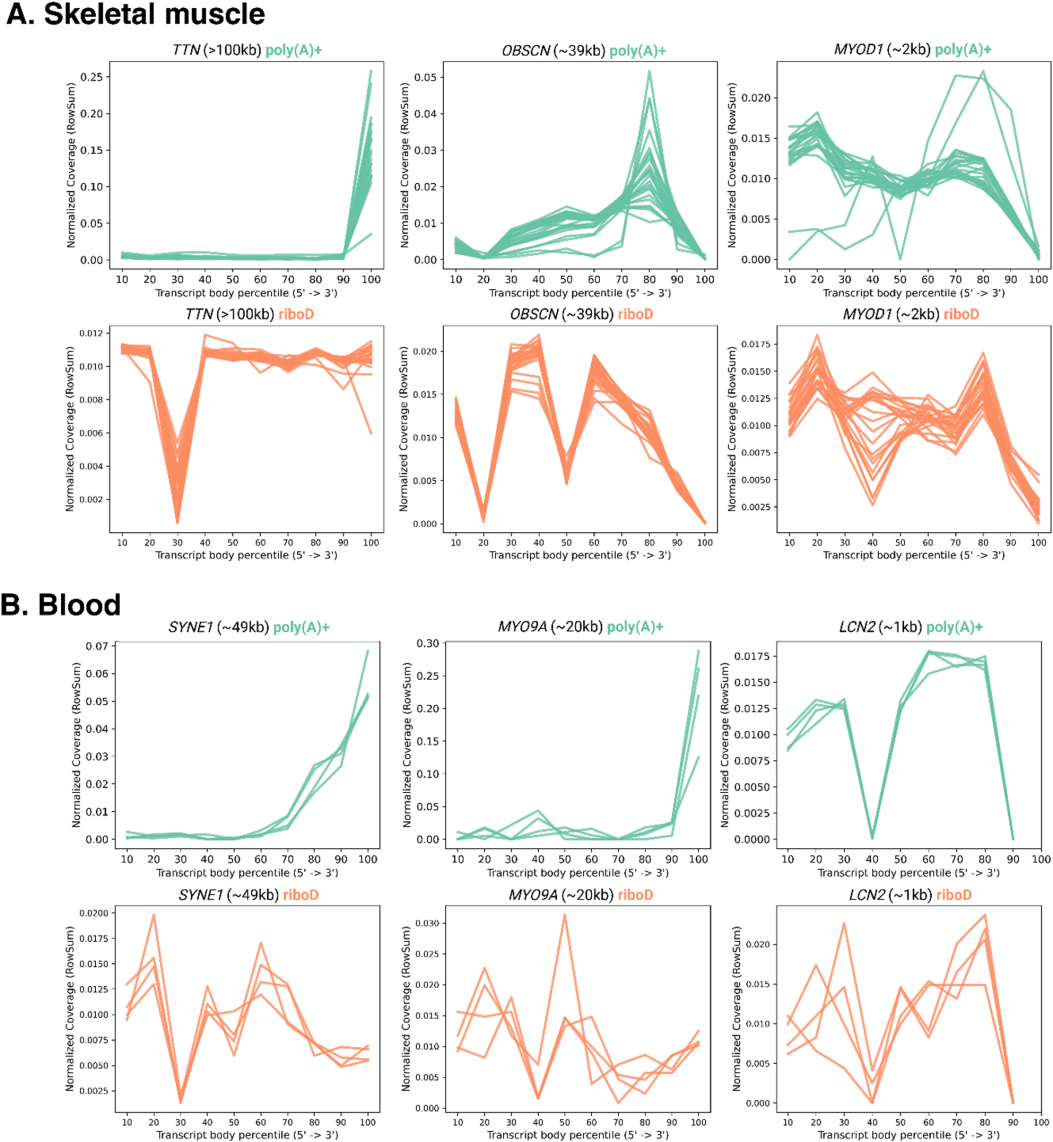
Normalized transcript body coverage profiles for riboD (rRNA depleted) and poly(A)+ selected genes of varying lengths. **A** Skeletal muscle samples. **B** Blood samples. Each line represents an individual sample within the respective cohort. Poly(A)+ libraries display a pronounced 3′-end bias for longer transcripts, whereas rRNA-depleted libraries do not

In order to illustrate how each library enrichment method influences the detection of individual genes, we compared the transcript body-coverage profiles (normalized by total coverage) of two muscle-function genes with long isoforms, *OBSCN* (~ 39 kb) and *TTN* (> 100 kb), to that of a gene with a substantially shorter isoform, *MYOD1* (~ 2 kb) (Fig. 2A). Poly(A)+ detection RNA-Seq from muscle biopsies displayed a clear decrease in read coverage toward the 5’ end, highlighting an overall strong 3’ end detection bias. In contrast, the rRNA depleted RNA-Seq mostly provided reduced 3’end bias compared with poly(A)+ selection (Fig. 2A**)**, with the exception of localized dips in the coverage due to low exon usage, particularly in *TTN *[30] and *OBSCN *[22].

In blood, transcript body coverage results for *SYNE1* (~ 47 kb) and *MYO9A* (~ 20 kb) exhibited a strong 3′ end bias and a very low coverage toward the 5’ end in poly(A) + RNA-Seq, whereas for the smaller transcript coded by gene *LCN2* (~ 1 kb) the coverage was more uniform (Fig. 2B**)**. In contrast, irrespective of transcription length, rRNA depleted RNA-Seq reads covered uniformly across the body of all three mentioned genes. The only exceptions were regions where exon usage differed among isoforms, which led to localized dips in the sequence read coverage. Figure 2 reveal a more uniform detection of splicing events such as exon usage in rRNA depleted libraries compared to poly(A)+ selected libraries for longer transcripts.

These transcript body coverage profile results are consistent with the 5’end − 3’end coverage ratio analysis (Supplementary 2 A & B; values for all annotated genes in Gencode v39 in Supplementary 1), which reflects the uniformity of read coverage across transcript ends. In our data, for large transcripts, rRNA depleted RNA-Seq exhibited coverage ratios closer to zero, indicating more uniform 5’-3’ end transcript coverage. In contrast poly(A) + RNA-Seq showed negative ratios for the larger transcripts reflecting its strong 3’ end bias (Supplementary 2 A).

### Ribodepletion RNA-Seq reads provides higher splice junction support

To directly assess the relationship between TL and splice junction detection, we checked both, the total number of splice junctions and the total read counts supporting those junctions across transcripts of varying length in SM and blood. For SM, we conducted a paired-analysis of four biopsies that were sequenced in both poly(A)+ enrichment and rRNA depletion RNA-Seq techniques. These four biopsies were derived from patients with a confirmed titin-associated myopathy diagnosis, enabling controlled within-sample comparisons of library enrichment techniques. For blood, we used all the technical replicates mentioned in the publicly available dataset (https://www.ncbi.nlm.nih.gov/sra/?term=SRP127360 SRP127360).

**Fig. 3:**
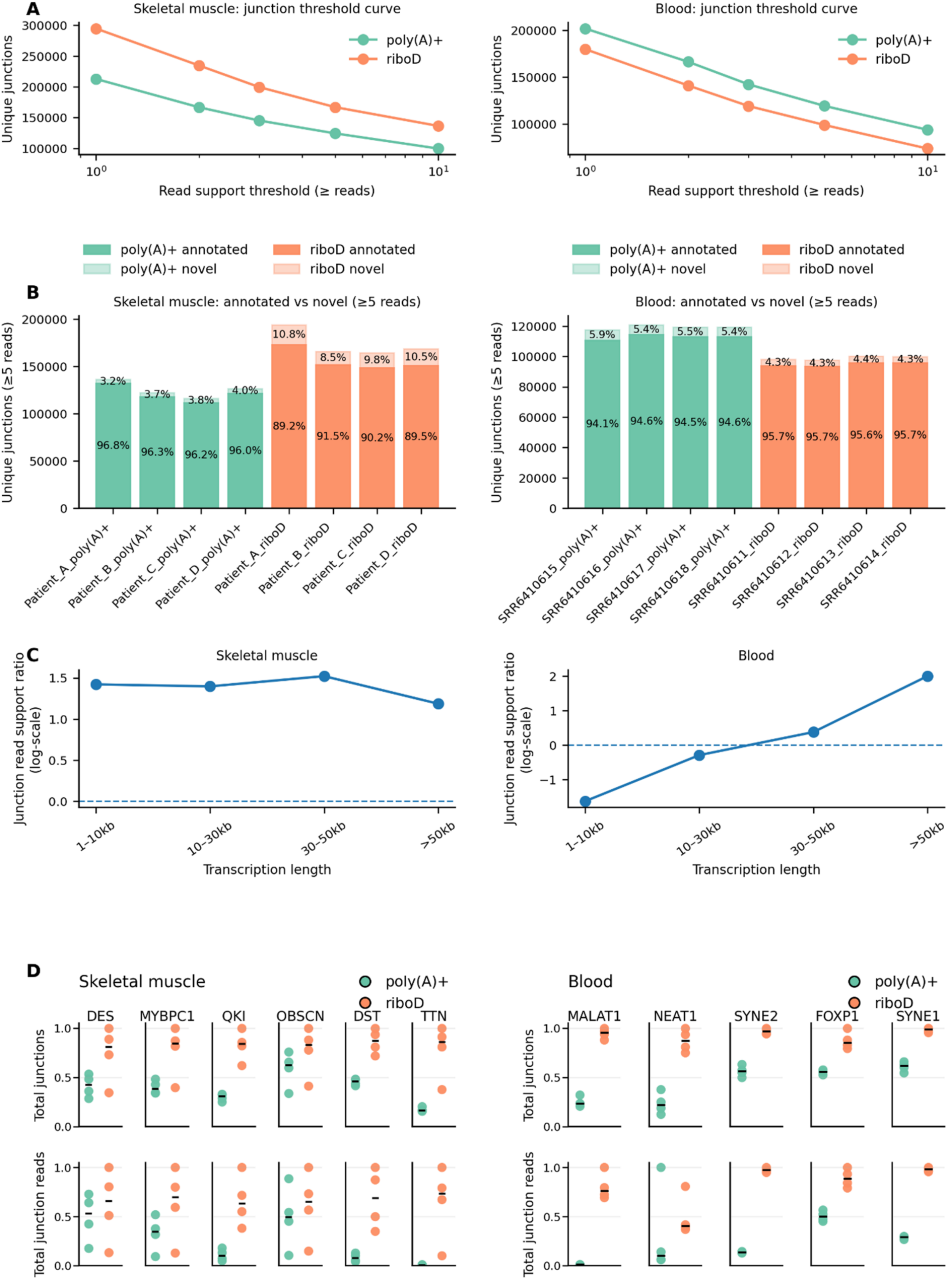
Transcription length-dependent differences in splice junction detection between poly(A)+ selection and rRNA depletion in skeletal muscle and blood RNA-Seq datasets. **A** Splice junction detection threshold curves for skeletal muscle (left) and blood (right), showing the number of unique splice junctions detected with minimum read-support threshold (≥ 1, ≥ 2, ≥3, ≥ 5, ≥10 reads). **B** Distribution of annotated versus novel splice junctions (≥ 5 supporting reads) in matched skeletal muscle samples (left) and blood samples (right). Percentages of annotated and novel junctions are indicated within bars. **C** Relationship between transcription length and total splice junction read support, expressed as log2(riboD / poly(A)+), across transcription length bins in skeletal muscle (left) and blood (right). Positive values indicate higher junction read support in rRNA-depleted libraries. In skeletal muscle (where rRNA is sequenced at higher depth to allow detection of similar levels of mRNA as in Poly(A)+ selection), rRNA depletion shows consistently higher junction support across all length bins, with modest variation across transcription lengths. In contrast, in blood (where rRNA depleted and Poly(A)+ selection were sequenced at similar depths), the junction support ratio increases with transcription length, indicating a stronger length-dependent divergence between enrichment strategies. **D** Gene-level comparison of normalized total splice junction counts (top panels) and total junction read support (bottom panels) in skeletal muscle (left) and blood (right). Values are scaled within each gene to facilitate within-gene comparison of enrichment techniques

It is important to note that sequencing depth differed between RNA-Seq libraries generated using the two enrichment methods of the paired-SM samples, with rRNA-depleted libraries sequenced at higher depth (~ 100 M) than poly(A)+ libraries (~ 52 M) (Supplementary 3). In contrast, read depth was comparable between libraries generated using the two enrichment methods in the blood cohort (~ 50 M) (Supplementary 3). Increased sequencing depth is commonly employed in rRNA-depleted datasets to compensate for their greater transcriptomic complexity, as these libraries retain total RNA (excluding rRNA), thereby increasing representation of pre-mRNA-derived intronic reads and non-polyadenylated RNA species, including many replication-dependent histone mRNAs, certain long non-coding RNAs, and circular RNAs^6^. Empirical comparisons indicate that substantially more reads are required for rRNA depletion than for polyA selection to achieve comparable mRNA levels and exonic coverage^6^. As expected, greater depth contributes to increased splice junction counts and higher read-support thresholds in skeletal muscle rRNA-depleted libraries (Fig. 3A-B, Supplementary 4). In contrast, the absence of additional sequencing depth in blood dataset resulted in rRNA-depleted libraries exhibited lower total junction counts at read-support thresholds (Fig. 3A-B, Supplementary 4). In skeletal muscle, where rRNA-depleted libraries were sequenced at greater depth to enable detection of mRNA levels comparable to poly(A)+ selection, rRNA depletion shows consistently higher junction support across all transcription length bins, with only modest variation across lengths (Fig. 3C, Supplementary 5). In contrast, in blood, where rRNA-depleted and poly(A)+ libraries were sequenced at similar depths, the junction support ratio increases with transcription length, indicating a stronger length-dependent divergence between enrichment strategies (Fig. 3C). Together, these observations indicate that, while splice junction metrics are strongly influenced by sequencing depth, transcription length-dependent performance can also be observed, particularly when similar sequencing depths are used for the two enrichment methods.

Importantly, these effects were not restricted to a single gene such as *TTN*. Gene-level analyses across additional transcripts of varying lengths (e.g., *DES* (2 kb), *MYBPC1* (7 kb), *QKI* (17 kb), *OBSCN* (39 kb), *DST* (48 kb) in skeletal muscle and then, *MALAT1* (8 kb), *NEAT1*(22 kb), *SYNE2* (32 kb), *FOXP1* (32 kb), *SYNE1*(47 kb) in blood) demonstrated consistently higher splice junction counts and splice junction read support in rRNA-depleted libraries (Fig. 3D, Supplementary 5). Together, these findings demonstrate that rRNA-depleted libraries progressively detect splice junctions more efficiently in longer transcripts compared to poly(A)+ libraries. They also show that, when sequenced at sufficiently high depth (as is commonly done to enable detection of comparable mRNA levels in rRNA-depleted versus poly(A)+ libraries), the improved detection of splice junctions in rRNA-depleted libraries is observed across genes of all transcription lengths (Fig. 3C).

These results also align with the observed transcript-body coverage patterns, where poly(A)+ libraries exhibit increased 3′ bias. Since long transcripts are more susceptible to incomplete coverage toward the 5′ end in poly(A)+ datasets, splice junctions located distal to the poly(A) tail may be underrepresented. In contrast, rRNA-depleted libraries retain more uniform 5’-3’ coverage, enabling more balanced splice junction detection across the full transcription length.

### Enrichment technique influences detected RNA biotypes and length-dependent expression estimates

**Fig. 4:**
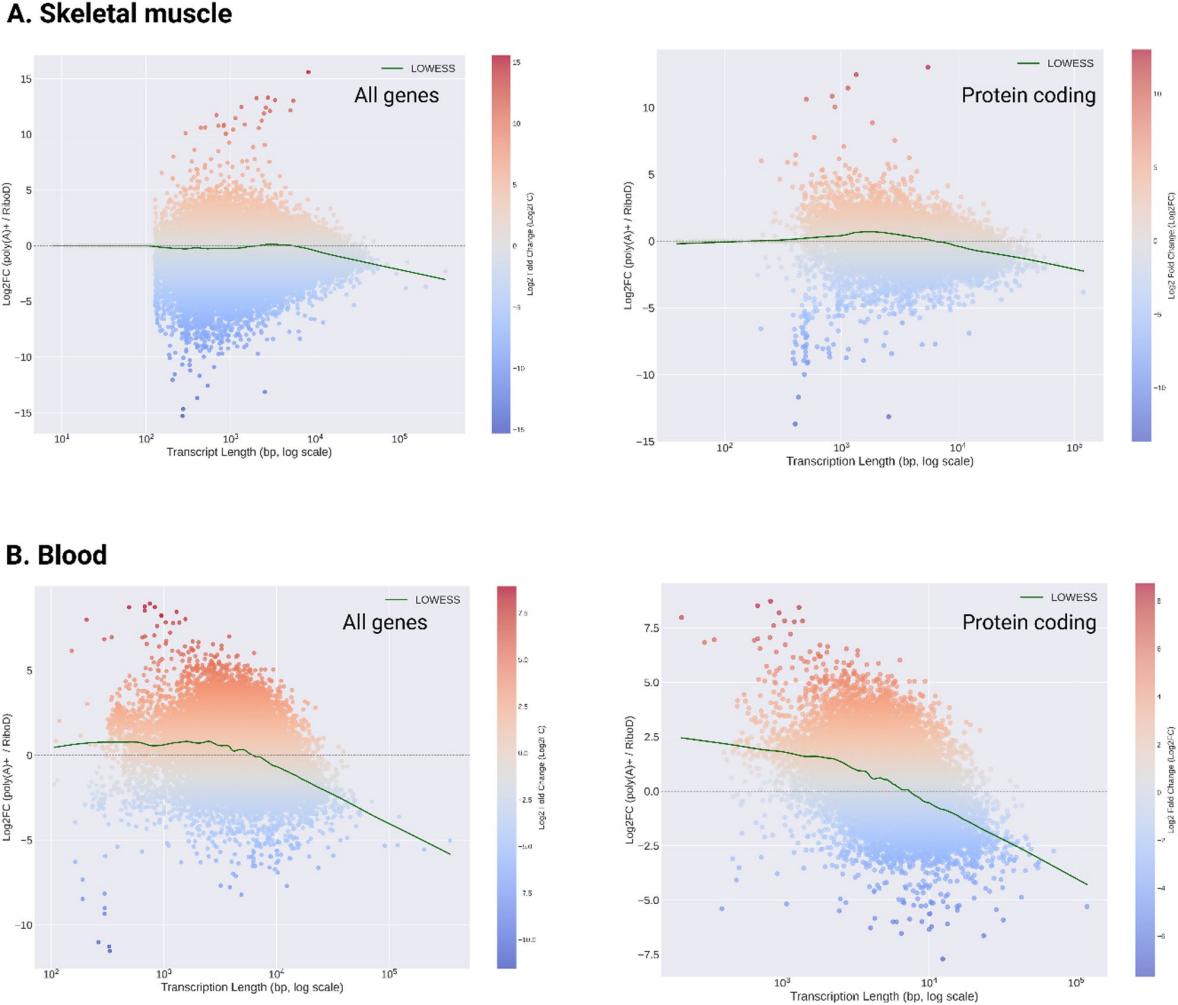
Gene expression detection patterns across transcription lengths and biotypes in poly(A)+ enriched and rRNA depleted RNA-Seq libraries. **A** Skeletal muscle. **B** Blood samples. Scatterplot of log_2_fold change (poly(A)+ / rRNA depletion) against transcription length (log_10_ scale). Each point represents a gene, colored by its average expression level (CPM). A LOWESS curve (green) highlights overall trends across lengths. Transcription length was plotted on a log10 scale, such that equal distances on the x-axis represent tenfold increases in length (e.g., 10^3^ bp is 1 kb, 10^4^ bp is 10 kb). Positive log_2_ values indicate higher expression in poly(A)+ libraries, whereas negative values indicate higher expression in rRNA depleted libraries. Equivalent analysis narrowed to protein-coding genes. The scatterplot illustrates length dependent protein coding expression. Scatter plots made using python

To characterize differences between enrichment techniques, we examined read distribution across genomic regions and the distribution of expressed gene biotypes in both skeletal muscle and blood datasets (Supplementary 2 C-D). Across both tissues, rRNA-depleted libraries showed increased representation of intronic reads and a modest increase in intergenic reads relative to poly(A)+ enrichment, as they constitute for a minute proportion of the detected RNAs in our data. Across both tissues, rRNA depletion revealed a higher proportion of lncRNAs and other non-protein coding RNA classes compared to poly(A)+ selected libraries (Supplementary 2 C-D). We further examined how gene expression values differ between the two library enrichment methods (Supplementary 6). We plotted the log₂ fold change between expression values obtained from poly(A) + RNA-Seq and those from rRNA-depleted RNA-Seq against TL for muscle and blood samples **(**Fig. 4, Supplementary 6). A LOWESS (Locally Weighted Scatterplot Smoothing) curve was fitted to illustrate the overall relationship between TL and fold-change values. In skeletal muscle (Fig. 4A), transcripts shorter than 5 kb, showed broadly similar expression levels between the two methods. In contrast, for longer transcripts, the distribution of values shifted toward negative log2 values, indicating higher expression detection in the rRNA-depleted dataset. The LOWESS curve demonstrated a pronounced downward trajectory at transcription length of 10^3^-10^4^, indicating that discrepancies in expression estimates between the two methods become more pronounced for transcripts longer than ~5 kb. This pattern is consistent with our earlier observations that rRNA-depleted RNA-Seq provides superior coverage and sensitivity for long transcripts. When the analysis was restricted only to protein-coding genes (Fig. 4), a similar length-dependent bias favoring rRNA depletion was observed. In blood, the trend was distinct. For genes with minimum CPM > 1 in each library enrichment group, the LOWESS curve shows a drastic shift below zero for transcription lengths above 5 kb, highlighted with a downward trajectory at transcription length of 10^3^-10^4^ (Fig. 4), A similar trend was observed when the analysis was restricted to protein-coding genes, with transcripts exceeding 10 kb showing higher expression detection in rRNA-depleted libraries.

A confirmatory analysis was performed to assess whether the observed length-dependent pattern was influenced by the quantification strategy. In addition to transcript-level NumReads estimates generated by Salmon and summarized to gene-level CPM values, we conducted an independent genome-alignment-based quantification to obtain gene-level counts. These counts were normalized using CPM and FPKM. Across both skeletal muscle and blood datasets, the same length-dependent differences between enrichment strategies were observed (Supplementary 7), indicating that the trend is robust to the choice of quantification pipeline and normalization method.

### rRNA depleted RNA-Seq facilitates improved detection and clinical interpretation of splice variants

**Fig. 5:**
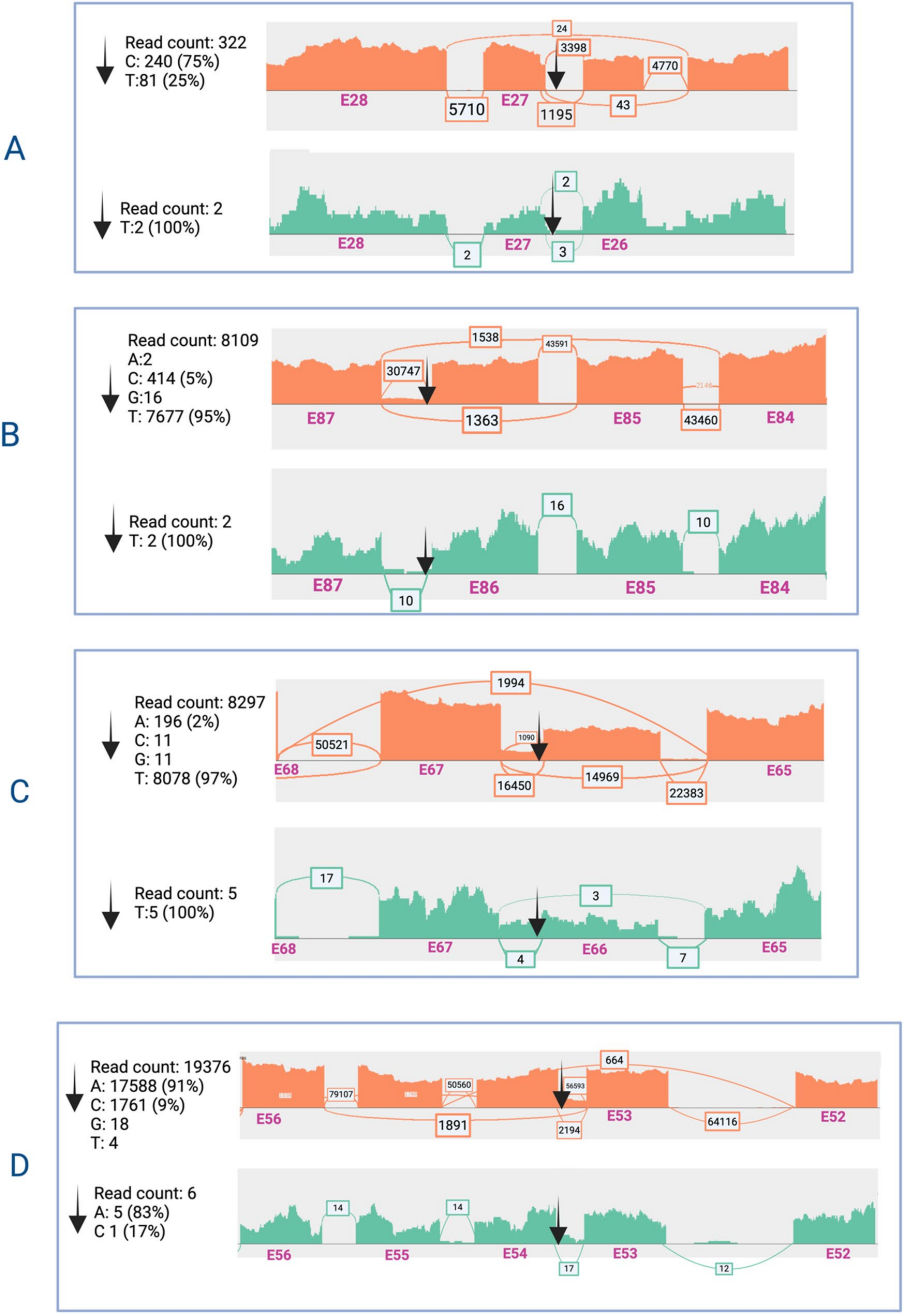
Sashimi plot for same skeletal muscle samples run in both poly(A)+ enrichment and rRNA depletion methods. Four human patient samples (**A**, **B**, **C**, **D**) with confirmed titinopathy were run in both RNA library approaches (top orange for rRNA depletion and bottom green for poly(A)+ enrichment). Snapshots from IGV sashimi showcase splice events for each library RNA run in each patient sample. Black arrows indicate the variant site. The splice junctions are coloured like the library group colour. The numbered box within each junction curve denotes the reads accounting for the splice junction. Exon numbers are labelled as E*nn* in pink. Biorender was used to include sashimi snapshots and mark arrows and read counts boxes for splice junctions

To evaluate how the choice of library enrichment strategy influences clinical interpretation and diagnostic sensitivity, we analyzed skeletal muscle biopsies from four patients with a confirmed titin-affected myopathy diagnosis using both rRNA depletion and poly(A)+ enrichment RNA-Seq methods. Each patient had a confirmed diagnosis of intronic variants in the *TTN* gene that caused splicing defects (More information in Supplementary 8). A two-tiered evaluation combining Integrative Genomics Viewer (IGV) [26] visualization and the DROP [27] RNA-Seq pipeline was employed to detect pathogenic variants and aberrant splicing events (Supplementary 2 E). Comparative analysis across the two sequencing methods indicated that rRNA-depleted RNA-Seq detected pathogenic variants with greater sensitivity and statistical confidence, whereas poly(A)+ enrichment RNA-Seq provided minimal coverage of the affected variant (Fig. 5; Supplementary 2 E). rRNA-depleted data consistently revealed patient-specific aberrant splice junctions, including complex exon-skipping events and activation of cryptic splice sites with statistical confidence (adjusted *p*-value < 0.01 in DROP pipeline) (Fig. 5; Supplementary 8). Notably, in this titinopathy cohort, where strong and previously characterized pathogenic splice variants are present, poly(A) + RNA-Seq failed to detect many novel and cryptic splicing events that were readily captured by rRNA-depleted RNA-Seq (Supplementary 8).

## Discussion

Over the last decade, RNA-Seq has become an increasingly important tool in both clinical diagnostics and biomedical research, owing to its ability to quantify gene expression patterns, detect splicing events and provide insights on transcriptome-wide alterations. Furthermore, it has expanded its role in understanding disease mechanisms and improving diagnostic yield [9, 23, 24, 27]. As RNA-Seq becomes increasingly incorporated into clinical settings [1, 31], selecting the appropriate enrichment strategy will be essential to maximize diagnostic yield. Early large-scale studies compared the two widely used library enrichment strategies, and demonstrated that RNA-Seq outcomes are strongly influenced by library preparation choices. Kapranov et al. systematically characterized RNA populations in human tissues beyond polyadenylated transcripts, at a transcriptome‑wide scale. Their work highlighted that a large set of sequences detected by rRNA depletion RNA-Seq, including intronic sequences and sequences from intergenic non-coding RNAs (with unknown functions) are poorly captured by poly(A)+ selection [10]. Their findings established that poly(A) + RNA-Seq provides a narrowed view of the polyadenylated transcriptome. However, their study focused on the extent to which RNAs with known or intergenic sequences of unknown function are detected. It did not focus on benchmarking short-read RNA-Seq library preparation techniques by assessing the influence of enrichment techniques on length-dependent transcript body coverage, splice junction support, or gene expression estimates. Barrett et al. [8] conducted head-to-head comparisons of poly(A)-based (SMART-seq V4) and rRNA depletion (SoLo Ovation) RNA-Seq in *Caenorhabditis elegans*, demonstrating notable advantages for rRNA depletion in the detection of noncoding RNAs, reduction of noise in lowly expressed genes, and more accurate quantification in long transcripts. However, the *C. elegans* genome differs significantly from that of humans, with notable differences in intron lengths, splicing complexity and gene-length distributions, and expression heterogeneity. Comprehensive evaluation of enrichment-dependent effects in human, patient-derived tissues, especially in the context of long, splice-complex transcripts in short read RNA-Seq has remained limited. 

Our study extends these foundational and model-organism studies to human skeletal muscle and blood transcriptomes using systematic short-read RNA-Seq workflows across independent and partially paired samples. Consistent with the earlier reports [6–10] rRNA depletion in our datasets captured a wider diversity of RNA biotypes, including higher representation of lncRNAs and other non-protein-coding transcripts relative to poly(A)+ selection (Supplementary 2 C & D). This confirms that the two enrichment techniques capture overlapping but non-identical RNA populations. Beyond RNA population differences, we observe that, in both groups of studied samples (skeletal muscle and blood), rRNA depletion libraries produce higher uniformity of reads along the transcript and, overall, improved transcript coverage. In contrast, poly(A)+ enriched libraries exhibited a pronounced 3′ end bias, particularly for transcripts longer than 5 kb, resulting in non-uniform coverage. Our splice junction analyses further corroborated these findings, demonstrating that rRNA depletion yields higher junction read support for long transcripts, supporting enhanced junction-level representation for long transcripts. Our data suggests an association between enrichment method and transcription length, characterized by a pronounced length-dependent divergence in splice junction support that is independent of the read depth differences observed in a subset of our data (i.e. skeletal muscle samples). Since rRNA-depleted libraries capture a broader RNA population, including pre-mRNA and intronic sequences, this may contribute to an increased detection of transcripts. However, in our analysis, splice junctions were defined using exon-exon junction annotations and reliable read-support thresholds, thereby not assessing unprocessed transcripts. Thus, the observed increase in junction read support for long transcripts reflects improved representation of splice junctions rather than solely intronic signal. Other key technical parameters, including library preparation kit and RNA integrity (RIN), batch, sex and disease status, mentioned in Supplementary 9 did not bias our findings. 

When evaluating relative expression estimates, we observed that log2 fold-change (poly(A)+ / rRNA depletion) shifted toward more negative values with increased transcription length, indicating higher normalized read counts for longer transcripts in rRNA-depleted libraries. It is important to note, that these expression differences should not be interpreted as evidence that one method is inherently more ‘accurate’ than the other. Rather, they reflect distinct RNA populations captured by each approach, together with RNA integrity. Poly(A)+ protocols enrich only transcripts that still carry an intact 3′ poly(A) tail and therefore reveal the abundance of polyadenylated transcripts at the timepoint of library preparation step. In contrast, rRNA-depletion captures both mature mRNAs and a broad range of additional RNA species, including pre-mRNAs and fragmented transcripts, thereby providing a different and wider view of the transcribed RNA pool. That said, longer transcripts show greater susceptibility to degradation and accumulate more fragmentation events under the same conditions [32, 33]. Consequently, poly(A)+ libraries may capture a subset of the 3’-end fragments from long genes, whereas rRNA-depleted protocols can detect any fragment regardless of polyadenylation status. This sampling asymmetry results in a length-dependent differences of long-gene expression values and produces the characteristic 3′ coverage bias observed in poly(A)+ datasets [8, 34]. 

From a diagnostic perspective, our findings offer major implications: improved coverage of long genes directly translates to enhanced detection of aberrant splicing and more reliable variant interpretation in diseases involving large transcripts, particularly those associated with *TTN*, *NEB*, and *OBSCN* which encode some of the longest mRNAs [19–22]. Long sarcomeric genes are significant targets in genetic testing for muscular dystrophies and cardiomyopathies, yet their complex architecture and large transcript size often hinder reliable read coverage [23, 35]. Visualization of coverage profiles further demonstrated that complex, multi-exon splicing events caused by pathogenic *TTN* intronic variants were robustly detected only in rRNA-depleted datasets. In contrast, these events were missed or underrepresented in poly(A)+ enrichment RNA-Seq, as denoted by our results using both IGV and the DROP pipeline. Although *TTN* and *OBSCN* provide striking examples of these effects because of their exceptional transcription length and splicing complexity, our findings are not limited to these genes alone. Genome-wide analyses across all annotated genes showed that the enrichment-dependent differences in transcript-body coverage, 5’-3’ bias, splice junction support, and expression become increasingly pronounced with transcription length. 

This study, despite the demonstrated advantages for rRNA depletion, is limited by the use of short-read RNA-Seq data, which cannot resolve full-length transcript isoforms or unambiguously reconstruct complex splicing patterns. Furthermore, short-read approaches risk missing rare or novel isoforms, particularly in large transcripts and are unable to reliably resolve loci containing long repetitive regions. Therefore, future work integrating long-read sequencing technologies, such as PacBio Iso-Seq or Oxford Nanopore, could complement our findings by enabling the detection of full-length and previously unannotated transcripts that may refine transcript models and isoform-level analyses [15, 16, 36–38]. Notably, several poly(A)-independent protocols have recently been adapted for long-read platforms, including Nanopore-based workflows [39, 40]. Because long-read sequencing methods capture a much broader fraction of the transcriptome, it remains unclear whether the length-dependent differences observed between poly(A) + and poly(A)-independent libraries persist in long-read total-RNA datasets, and if so, to what extent. In addition, most comparisons between poly(A) + and rRNA-depleted libraries in this study were conducted on independent skeletal muscle cohorts rather than paired samples from the same individuals. Given the known biological variability in gene expression and splicing among individuals, including possible sex- and disease-associated effects, this may introduce inter-individual variability rather than enrichment technique alone. To help address this limitation, we included a paired analysis of four skeletal muscle samples, as well as an independent blood dataset where both library enrichment techniques were generated from aliquots of the same pooled RNA source. The similarity of the observed trends across independent cohorts, paired samples, and the pooled blood dataset enhances the robustness of the observed length-dependent effects.

Together, our analyses show that while poly(A)+ enrichment remains suitable for profiling polyadenylated transcript abundance in standard gene expression studies, rRNA-depleted RNA-Seq enhances the detectability of splice junctions by providing higher read support and more uniform transcript-body coverage, especially in genes with long and splice-heavy transcripts.

## Conclusion

Our data demonstrate that rRNA depleted RNA-Seq provides improved coverage, sensitivity, uniformity, transcript integrity and statistical confidence, enabling detection of splicing aberrations and enhancing variant interpretation. As RNA-Seq becomes increasingly central to molecular diagnostics, careful selection of library enrichment strategies is essential to maximize diagnostic yield and improve variant interpretation. Rather than indicating methodological correctness, the differences observed between poly(A)+ selection and rRNA depletion highlight the importance of selecting an enrichment method relevant to the biological question and clinical context. In particular, rRNA depletion may be advantageous in contexts requiring more uniform coverage, enhanced splice junction detectability and splice-aware variant interpretation, especially for longer transcripts, whereas poly(A)+ selection remains appropriate for studies focused on mature and polyadenylated mRNA expression, particularly for transcripts of average or shorter length (< 5 kb). By clarifying the interpretative consequences of commonly used RNA-Seq enrichment methods in patient-derived tissues, this study provides a clear framework for informed library preparation choice and suitable downstream analysis in both research and diagnostic settings.

## Materials and methods

### In-house RNA sequencing data

Forty-six different patient-derived skeletal muscle samples were selected for RNA sequencing for each enrichment strategies: twenty-three for rRNA depletion and twenty-three for poly(A)+ selection. Additionally, four samples were processed using both enrichment techniques, with each of these samples subjected to both rRNA depletion and poly(A)+ selection. In supplementary files, these samples are labelled as ‘polyA_1–23’ and ‘riboD_1–23’ for skeletal muscle cohorts, and ‘Patient A-D’ for paired skeletal muscle samples. Muscle tissue were homogenized in-house using SpeedMill PLUS (Analytik Jena AG, Germany). RNA was extracted with Qiagen RNeasy Plus Universal Mini Kit (Qiagen, Hilden, Germany) according to the manufacturers’ instructions. The RNA RIN value for samples in each skeletal muscle sample cohort (rRNA depletion and polyA+ selection), and the four samples run in both enrichment techniques had average RIN 7. Total RNA-Seq strand-specific libraries were prepared using the Illumina Ribo-Zero Plus rRNA Depletion Kit (Illumina, Palo Alto, CA, USA) at the Oxford Genomics Center, University of Oxford, Oxford, United Kingdom. Sequencing was performed on NovaSeq 6000 (Illumina), generating approximately 110 million paired-end reads per sample, with a total read length of 302 bp. For poly(A)+ enrichment, the NEBNext Ultra II Directional RNA Library Prep kit (E7760) for Illumina (NEB, Beverly, MA, USA) was used to prepare strand-specific RNA-Seq libraries. Libraries were multiplexed and sequenced on HiSeq4000 (Illumina, CA, USA), and approximately 70 million paired-end reads were produced, also with a total read length of 302 bp. All libraries were generated using random-primed first-strand cDNA synthesis. Strand specificity in both approaches was achieved via dUTP incorporation during second-strand synthesis.

### Public RNA sequencing data

Publicly available blood RNA-Seq data were obtained from the Sequence Read Archive (SRA) under accession number SRP127360 (labelled as SRR sample specific IDs in supplementary files). This dataset includes blood samples processed using both rRNA depletion and poly(A)+ enrichment [6]. However, prior to the analysis, and in consultation with the data curator and maintainer, we updated the sample annotation to correct an identified discrepancy. The finalized annotation for both blood and skeletal muscle data with read distribution by genomic position are provided in the supplementary materials (Supplementary 2 C).

### Quality control and read alignment

Raw sequencing reads were subjected to quality control using FastQC [41] to assess base quality scores, GC content, and adapter contamination. All samples exhibited high Phred quality scores across read lengths and were considered for further analysis. Reads were aligned to the human reference genome GRCh38.p13 using STAR v2.7.0a [42] following the two-pass mapping pipeline. The STAR genome index was generated from the Gencode v39 annotation, comprising 61,533 isoforms. To check how mapped reads were distributed over genomic regions (exonic, intronic and intergenic), the read_distribution.py command was used from ReSEQC.

### Splice junction analyses

To reduce biological variability, splicing junction analyses were performed using the four skeletal muscle samples sequenced in both enrichment techniques, as well as the blood dataset. Exon-exon junctions were extracted from BAM files using regtools [43] with Gencode v39 annotation. For each sample, we quantified the total number of uniquely detected junctions and cumulative junction read support. Junction counts were aggregated to gene-level metrics based on transcript-to-gene mapping. To ensure robust comparison, analyses were restricted to highly expressed genes (CPM > 10 in ≥ 70% of samples within each cohort). The relationship between transcription length and junction read support was assessed across transcription length bins, and enrichment-dependent differences were quantified using log2(riboD / poly(A)+) ratios. Junction read support values were max-scaled normalized in each cohort for cross-sample comparison. To evaluate junction saturation and annotation, ReSEQC tools junction_annotation.py and junction_saturation.py were used on mapped BAM files.

### Read quantification and transcription length 

Transcript-level quantification was obtained with Salmon [44],The resulting transcript per million (TPM) counts were then aggregated by sum to achieve gene-level counts. Genes with TPM > 1 were used for analysis, ensuring that only sufficiently expressed transcripts were included in the gene body coverage assessment. This filtering was performed separately for each tissue type to reflect tissue-specific expression profiles. To enable accurate comparisons between library enrichment approaches, these gene-level counts were converted to counts per million (CPM) and normalized for sequencing library size (Supplementary 6). For each gene, the log-scaled relative average expression achieved by rRNA-depleted RNA-Seq to the average expression achieved by poly(A) + RNA-Seq was measured. A confirmatory analysis was also performed by acquiring gene-level read summarization using HT-Seq [45] and performing the same pipeline with CPM and FPKM normalization.

Gene length was defined as the transcription length (TL), calculated by summing the lengths of all annotated exons across all transcripts corresponding to each gene in Gencode v39 annotation (Supplementary 10).

### RSeQC analyses and read coverage uniformity

To assess gene body coverage (GBC), the geneBody_coverage.py tool from the RSeQC package [46] was utilized. GBC analysis was performed on the mapped BAM files, restricted to the genes based on tissue-specific expression profiles. This tool divides each transcript into 100 equally sized bins along the 5’-3’ end and calculates read coverage within each bin, enabling the evaluation of coverage uniformity across transcripts (Raw coverage values for *TTN*,* OBSCN*,* MYOD1* for each enrichment technique are mentioned in Supplementary 11). The coefficient of variation (CV) was measured across the 100 bins across the length of each gene and log scale. A low CV value indicates a more uniform read distribution, whereas a higher CV indicates a less uniform read distribution. To further evaluate the 5’ and 3’ end coverage biases, raw read coverage for the first and last 20% of each transcript were extracted from the bin read coverage values. Their 5’-end to 3’-end ratio was calculated, for specific transcripts, where values closer to zero indicate more balanced coverage across the transcript body. For plotting the transcript body coverage profile of each gene, the raw transcript coverage values were normalized to the sum of the values within each sample.

### DROP pipeline to detect aberrant splicing effects

Four SM samples with confirmed diagnosis were processed using both rRNA depleted and poly(A)+ enrichment methods. The aberrant splicing module (version 1.4.0) in DROP [27] was used to detect pathogenic variants and aberrant splicing. The recommended cohort size is 30 samples for statistical significance, we ran DROP for these four SM samples as a part of larger cohorts sharing the same technical aspects of library preparation and sequencing facility (Supplementary 8). For the rRNA depleted samples we had a cohort of 53 and respectively for the poly(A)+ enriched the samples were part of a 96-sample cohort. We evaluated if the predicted splicing events were captured by the aberrant splicing module using the default settings. When interpreting the results, we checked events significant either by their original adjusted *p*-value or by a Bonferroni-corrected *p*-value calculated only across muscle related genes. This DROP pipeline was executed using the publicly available implementation provided by the developers, following the official documentation and recommended settings. No custom modifications were introduced to the workflow.

### Visualization of transcript body coverage

IGV was used to generate locus-specific coverage snapshots of pathogenic variant sites and assess aberrant splicing patterns in skeletal muscle biopsy samples. JBrowse2 [47] was used to visualize transcript body coverage across and provide qualitative assessment of 5’-3’ end coverage. The commands used for JBrowse2 are mentioned in Supplementary 12.

### The use of generative AI and AI-assisted technologies in the writing process

For preparation of this work, the authors have used ChatGPT to correct the grammar and proofread the text. After applying ChatGPT, the authors reviewed and further modified the text. The authors take full responsibility for the content in this publication.

## Supplementary Information

Supplementary Material 1.

Supplementary Material 2.

Supplementary Material 3.

Supplementary Material 4.

Supplementary Material 5.

Supplementary Material 6.

Supplementary Material 7.

Supplementary Material 8.

Supplementary Material 9.

Supplementary Material 10.

Supplementary Material 11.

Supplementary Material 12.

## Data Availability

RNA sequencing data for human blood samples were used from SRA (SRP127360). RNA sequencing data human skeletal muscle biopsies are protected under GDPR principles. The workflow chart and additional code used in this study are mentioned in Supplementary file 12.
