# Supplementary material for: Poly(A)+ selection limits detection of long and alternatively spliced transcripts compared with rRNA depletion in RNA-Sequencing
Source: BMC Genomics. 2026 May 13;27:591. doi: 10.1186/s12864-026-12944-z (PMC13339413; doi:10.1186/s12864-026-12944-z)
Supplement: Supplementary file 2 — Supplementary Material 2. [file 12864_2026_12944_MOESM2_ESM.pdf]

Supplementary 2

A. 5’-3’ end coverage assessment

Skeletal muscle: *TTN* (>100kb) , *OBSCN* (~39kb), *MYOD1* (~2kb)

Blood: *SYNE1* (~47 kb), *MYO9A* (~20 kb), *LCN2* (~1kb)

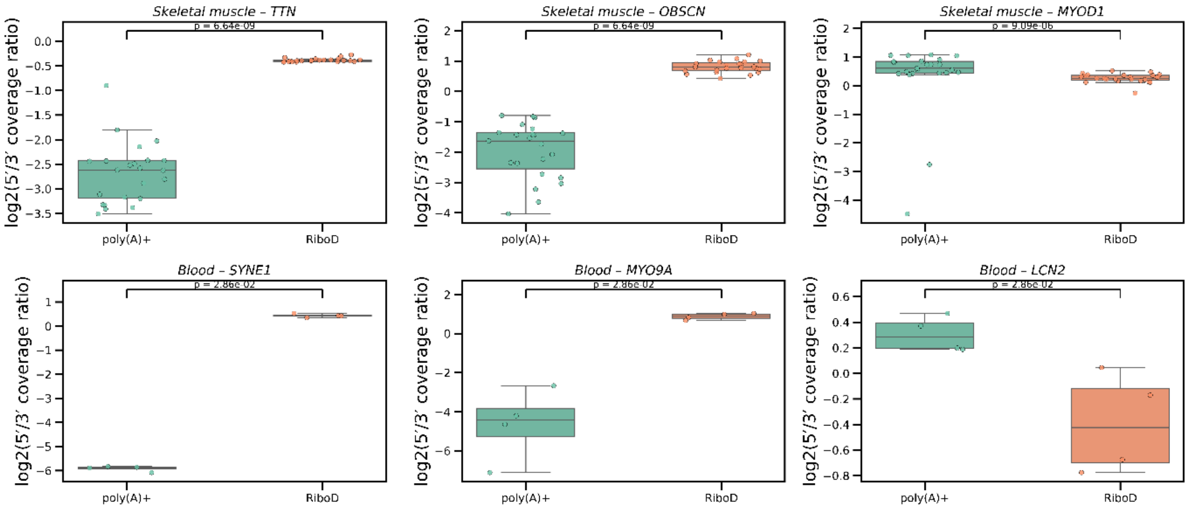

B. **Jbrowse2 images of transcript coverage (Blood)**

SYNE1 Chromosome 6: 152,121,687-152,637,801 reverse strand.

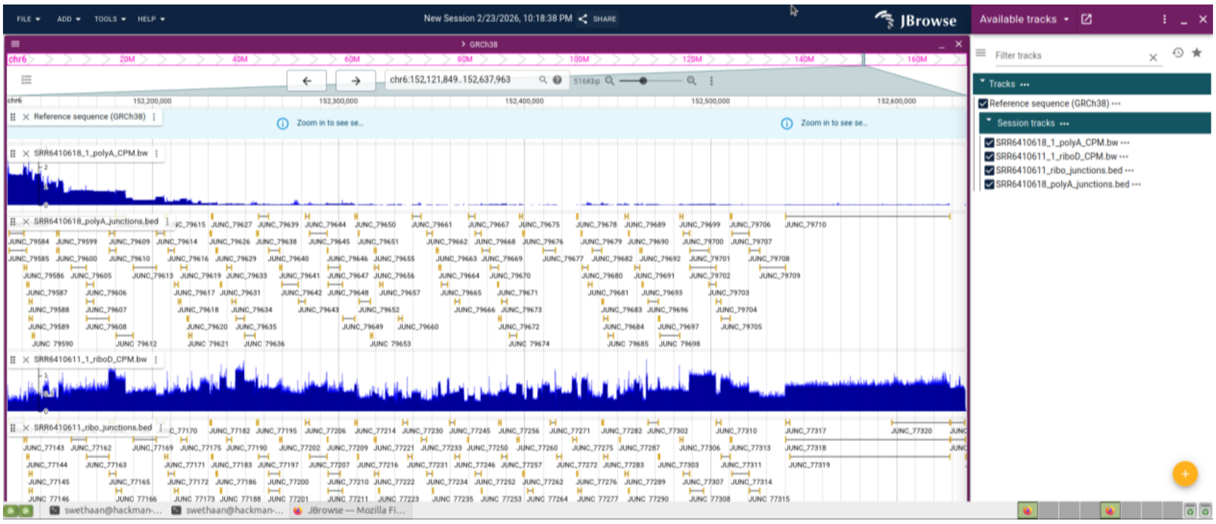

SYNE2 Chromosome 14: 63,761,899-64,226,433 forward strand.

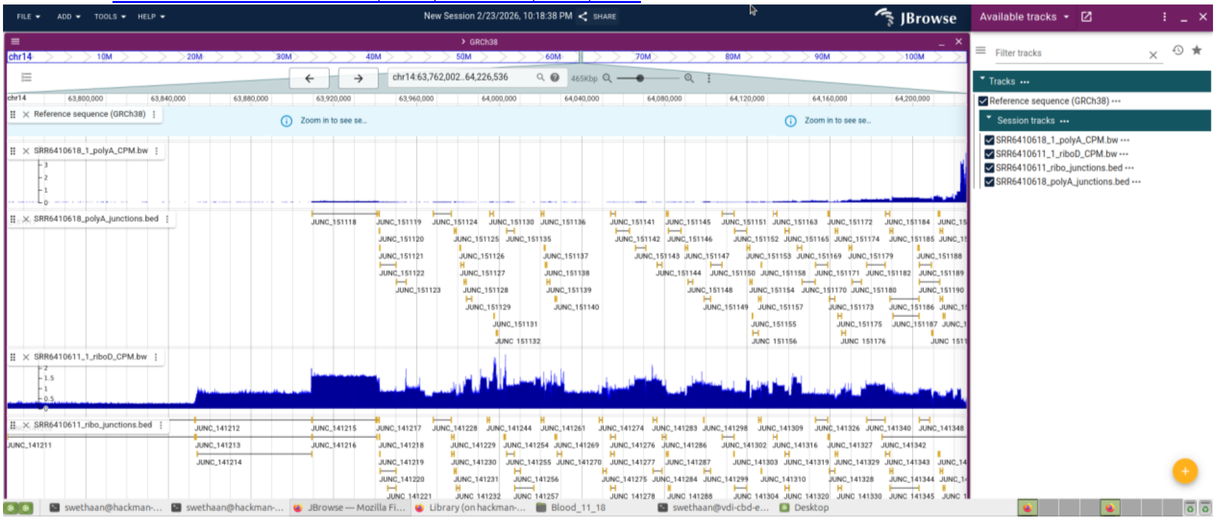

FOXP1 Chromosome 3: 70,954,693-71,584,009 reverse strand.

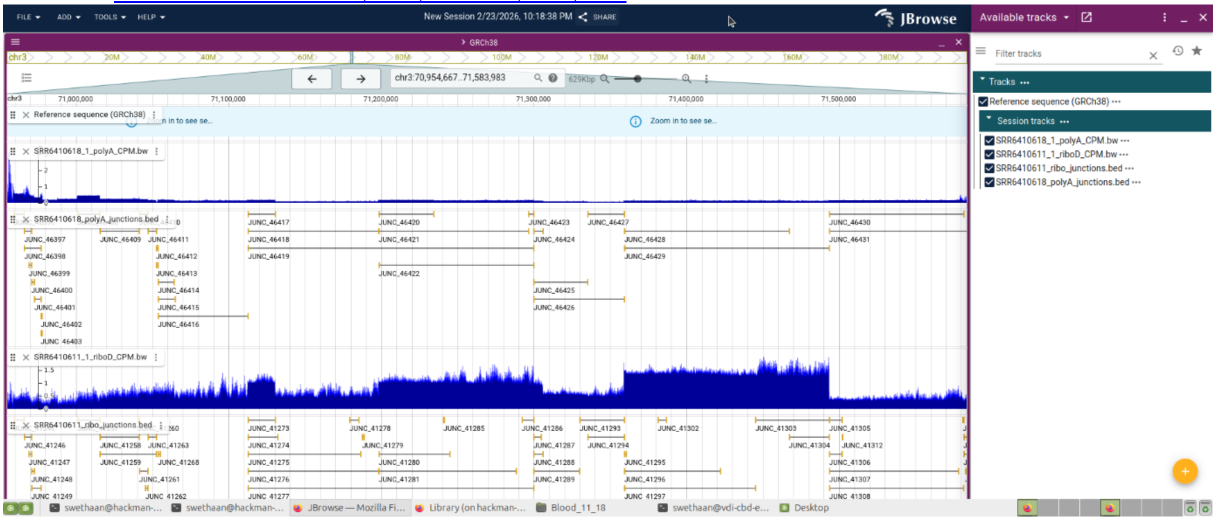

ACAB Chromosome 12: 109,116,587-109,268,226 forward strand.

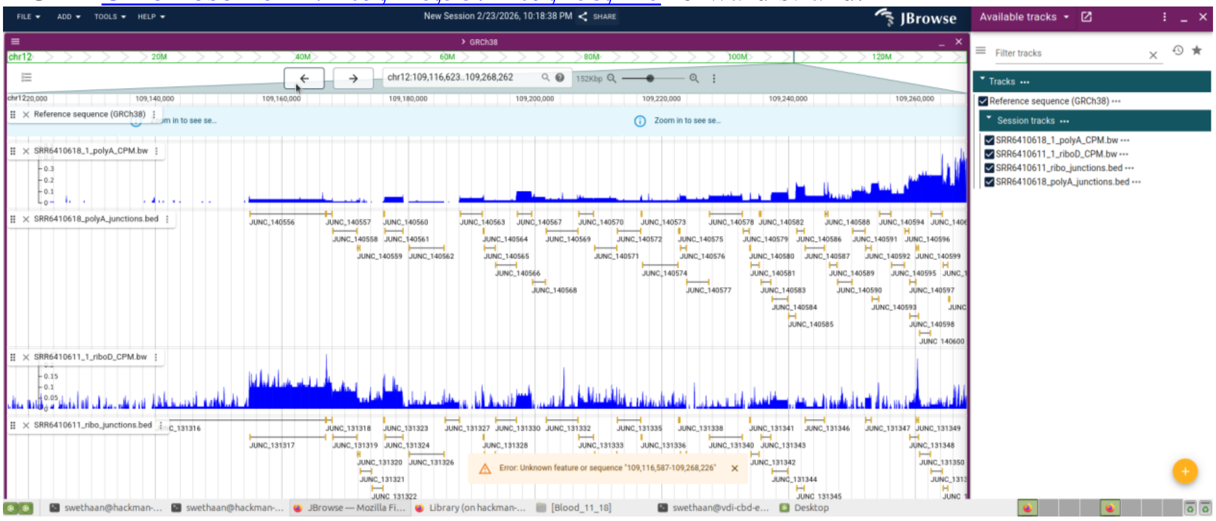

ALDH3B1 Chromosome 11: 68,008,563-68,029,282 forward strand.

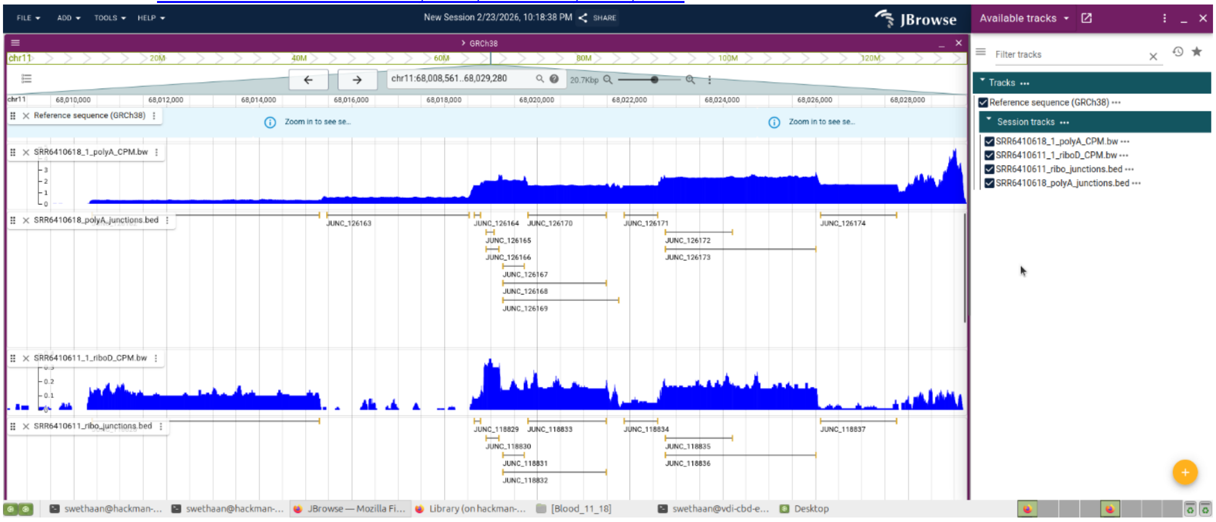

HELLPAR Chromosome 12: 102,197,585-102,402,596 forward strand.

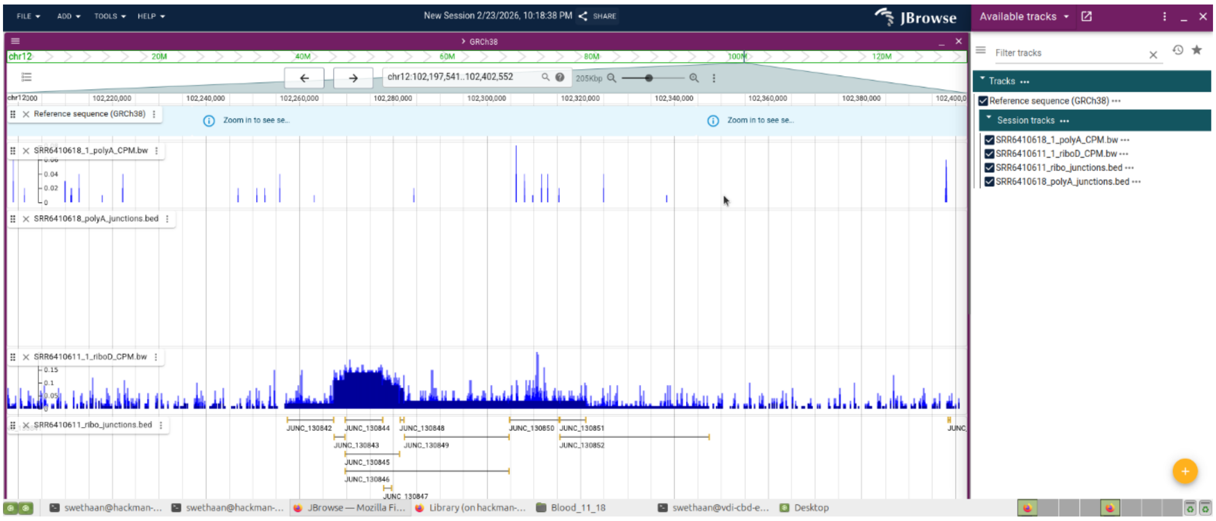

C. Read distribution by genomic region (exonic, intronic, intergenic)

Blood dataset

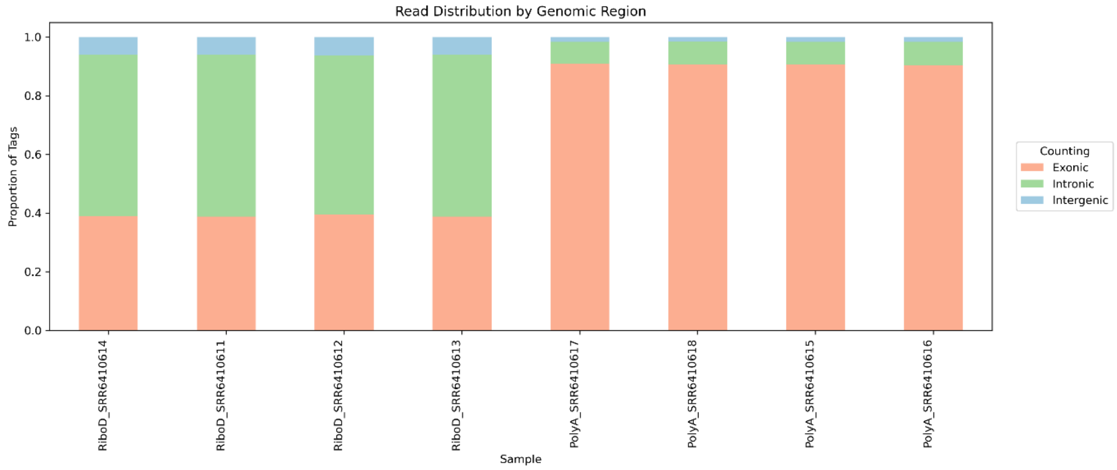

Skeletal muscle dataset

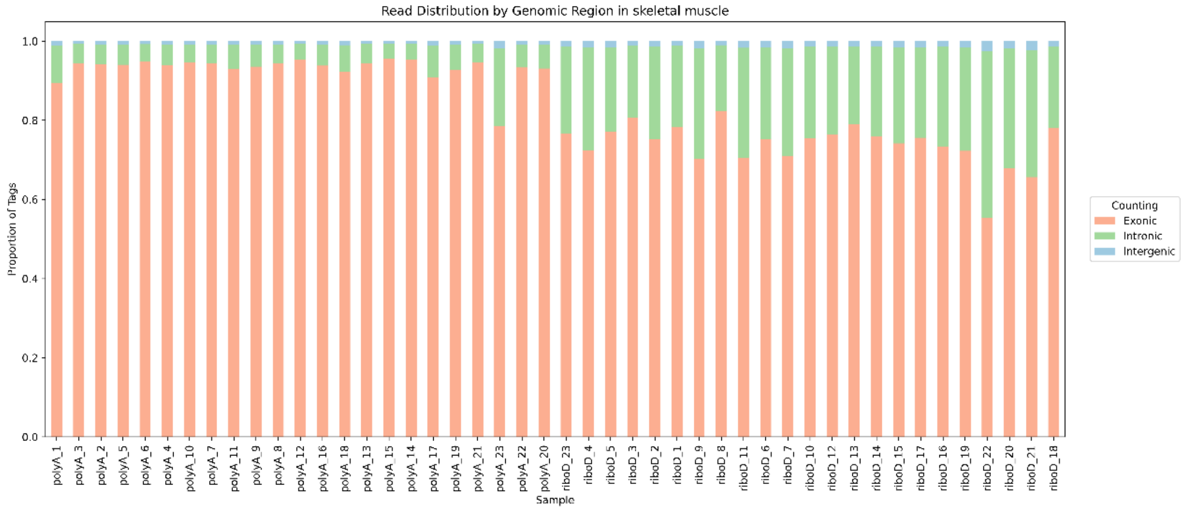

D. Sample biotype distribution based on expression data

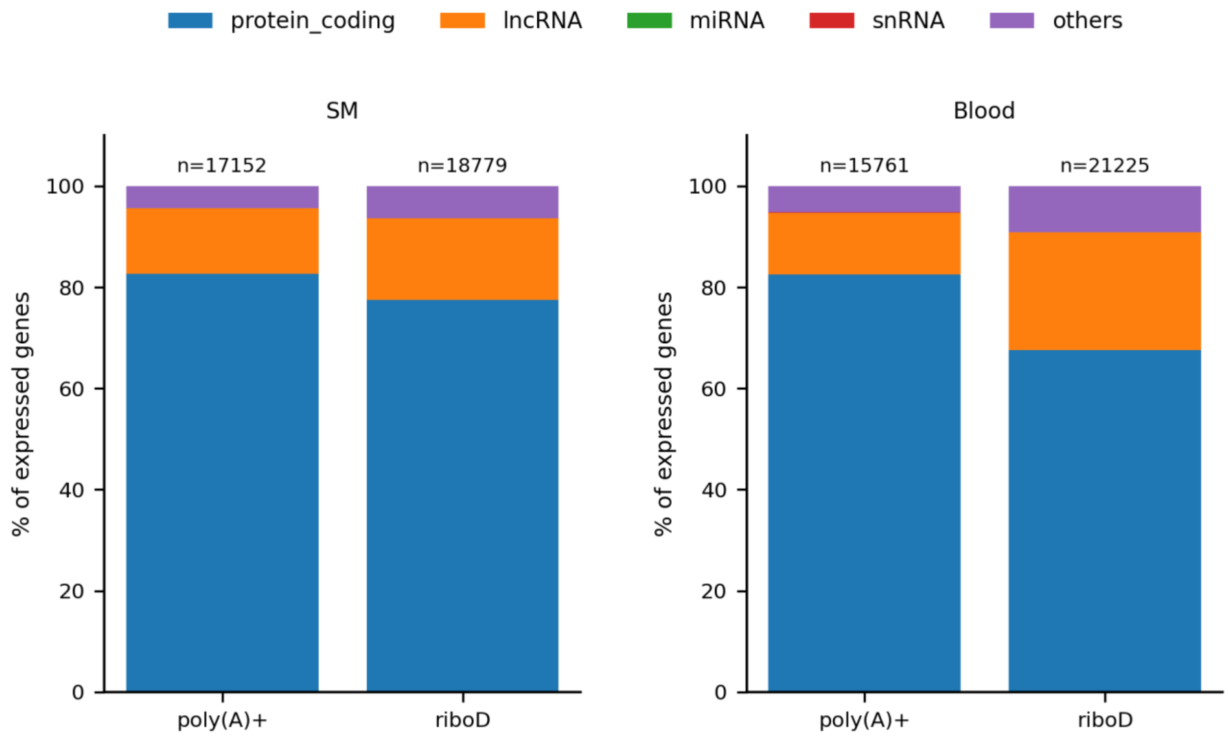

Tissue	Group	Category	N_genes	Total_expressed_genes	Pct_genes
SM	polyA	protein_coding	14172	17152	82.62593283582089
SM	polyA	lncRNA	2221	17152	12.948927238805972
SM	polyA	miRNA	1	17152	0.005830223880597014
SM	polyA	snRNA	3	17152	0.017490671641791043
SM	polyA	others	755	17152	4.4018190298507465
SM	riboD	protein_coding	14551	18779	77.48548911017626
SM	riboD	lncRNA	3033	18779	16.15101975611055
SM	riboD	miRNA	1	18779	0.00532509718302359
SM	riboD	snRNA	6	18779	0.03195058309814154
SM	riboD	others	1188	18779	6.326215453432026
Blood	polyA	protein_coding	13004	15761	82.50745511071632
Blood	polyA	lncRNA	1929	15761	12.239071124928621
Blood	polyA	miRNA	0	15761	0.0
Blood	polyA	snRNA	4	15761	0.025379100310893975

Blood	polyA	others	824	15761	5.22809466404416
Blood	riboD	protein_coding	14352	21225	67.61837455830388
Blood	riboD	lncRNA	4924	21225	23.199057714958776
Blood	riboD	miRNA	3	21225	0.014134275618374558
Blood	riboD	snRNA	15	21225	0.0706713780918728
Blood	riboD	others	1931	21225	9.097762073027091

E. Titinopathy patient information with IGV explanation

Patient A: TTN(NM_001267550.2):c.4646-1G >A

An intronic variant was identified in intron 26 of 362 in both poly(A)+ (supported by 2 reads) and rRNA depleted (supported by 322 reads) RNA-Seq. Consistent with the acceptor loss outcome, only rRNA depleted RNA revealed multiple aberrant splice events. These included the combined skipping of exons 26 and 27 (supported by 24 reads), occurrence of a cryptic acceptor site resulting in the loss of first 14 nucleotides in exon 27, supported by 1195 reads), and the skipping of exon 26 (43 supporting reads).

Patient B: TTN(NM_001267550.2):c.25063+1G>A

An intronic variant was identified in intron 86 of 362 in both poly(A)+ (supported by 2 reads) and rRNA depleted (supported by 8109 reads) RNA-Seq. The variant causes the loss of the donor site and only rRNA depleted RNA-Seq revealed clear evidence of aberrant splicing junctions, including the skipping of exon 86 (1363 supporting reads) and the combined skipping of exons 85 and 86 (1538 supporting reads).

Patient C: TTN(NM_001267550.2):c.19426+2T>A

An intronic variant was detected in intron 66 of 362 in both poly(A)+ (supported by 5 reads) and rRNA depleted (supported by 8297 reads) RNA-Seq. Although the intron retention is observed in both RNA libraries, there are more reads supporting the intronic region in rRNA

depleted RNA-Seq compared to ploy(A)+. Furthermore, only the rRNA depleted RNA-Seq demonstrated clear aberrant splicing junctions in IGV. Specifically, the skipping of exon 66 was supported by 14969 reads, whereas there are only 3 reads supporting that aberrant junction in poly(A)+ RNA-Seq data. In addition, rRNA depleted RNA-Seq revealed an alternative aberrant junction supporting the skipping of exons 66 and 67 by 1994 reads.

Patient D: TTN(NM_001267550.2):c.15776-1G>T

An intronic variant was identified in intron 53 of 362 in both poly(A)+ (supported by 6 reads) and rRNA depleted (supported by 19376 reads) RNA-Seq data. The variant causes the loss of the acceptor site and only rRNA depleted RNA-Seq clearly indicated the alternative splicing events (***Figure 2 D)***, including the skipping of exons 53 and 54 (664 supporting reads) and the skipping of exons 54 and 55 (1891 supporting reads).
