# Supplementary material for: Poly(A)+ selection limits detection of long and alternatively spliced transcripts compared with rRNA depletion in RNA-Sequencing
Source: BMC Genomics. 2026 May 13;27:591. doi: 10.1186/s12864-026-12944-z (PMC13339413; doi:10.1186/s12864-026-12944-z)

Supplementary 7

Blood dataset for gene-level counts with minimum CPM > 1 in each enrichment group:

log2FC (mean_CPM_poly(A) / mean_CPM_riboD)

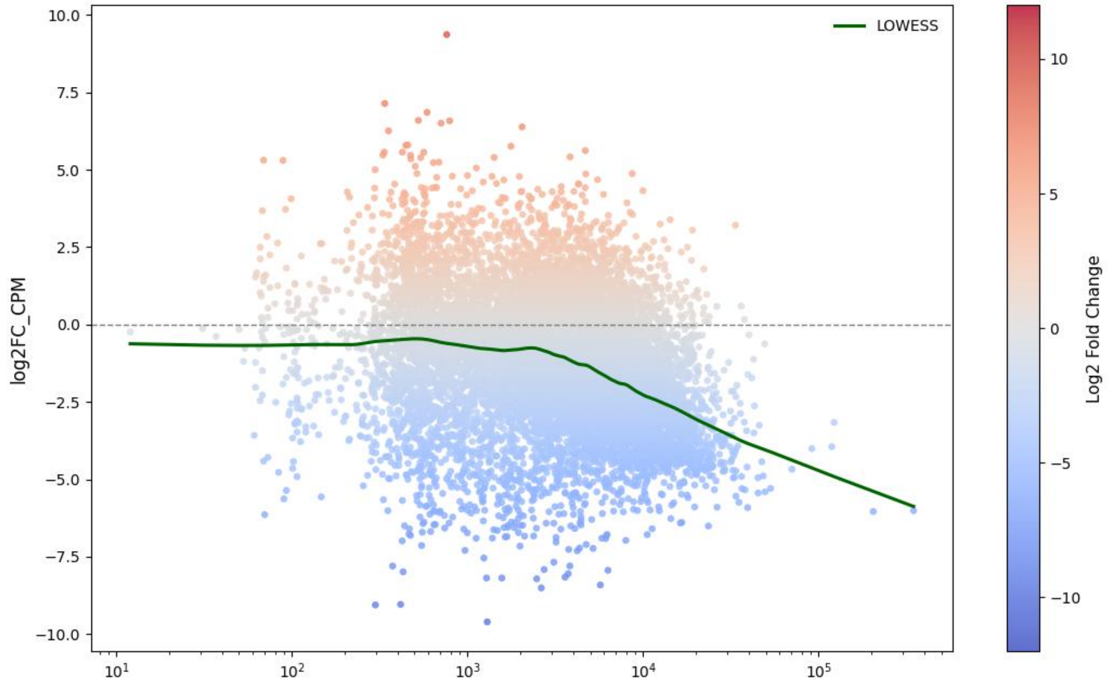

Blood dataset for all genes: log2FC (mean_FPKM_poly(A) / mean_FPKM_riboD)

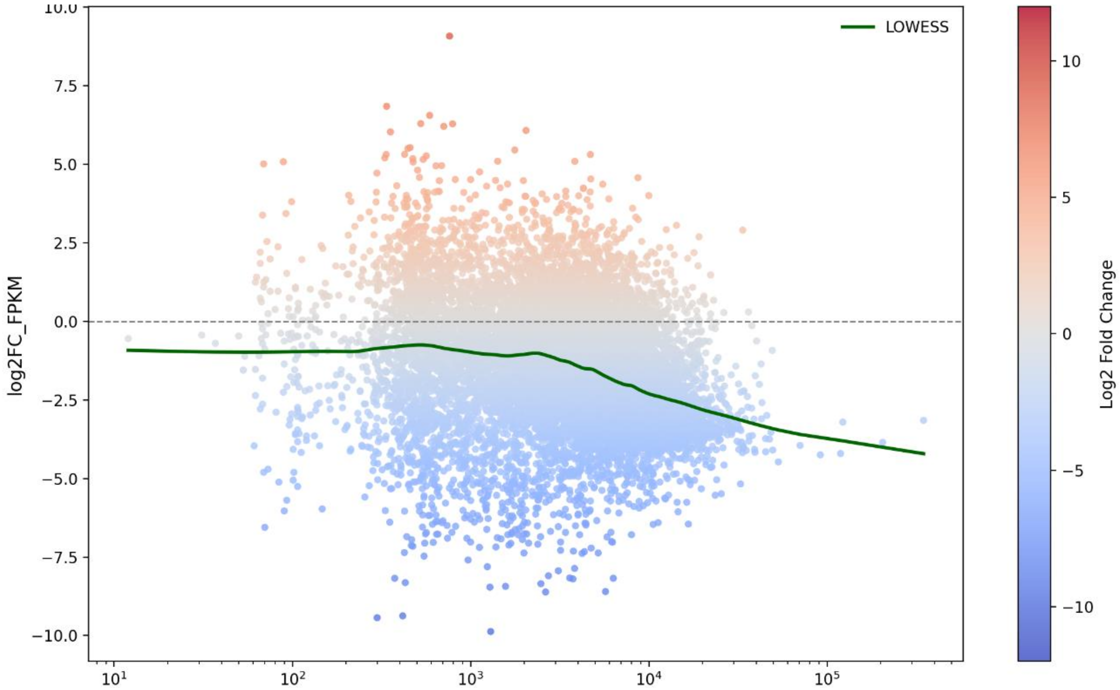

Skeletal muscle dataset for all genes: log2FC (mean_CPM_poly(A) / mean_CPM_riboD)

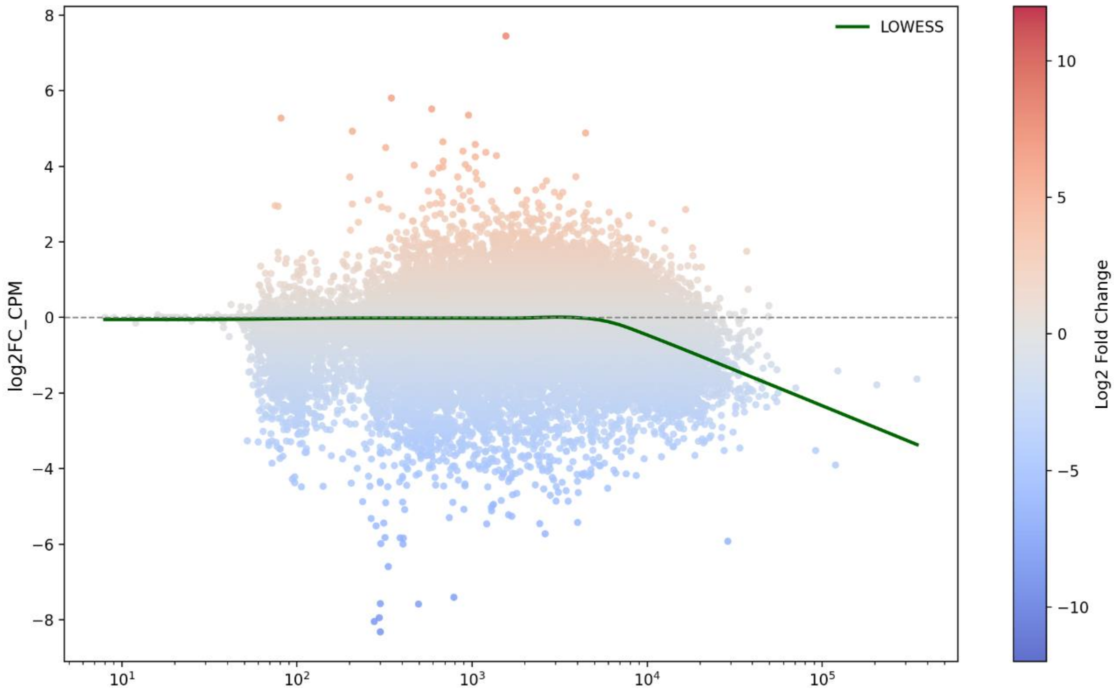

Skeletal muscle dataset for all genes: log2FC (mean_FPKM_poly(A) / mean_FPKM_riboD)

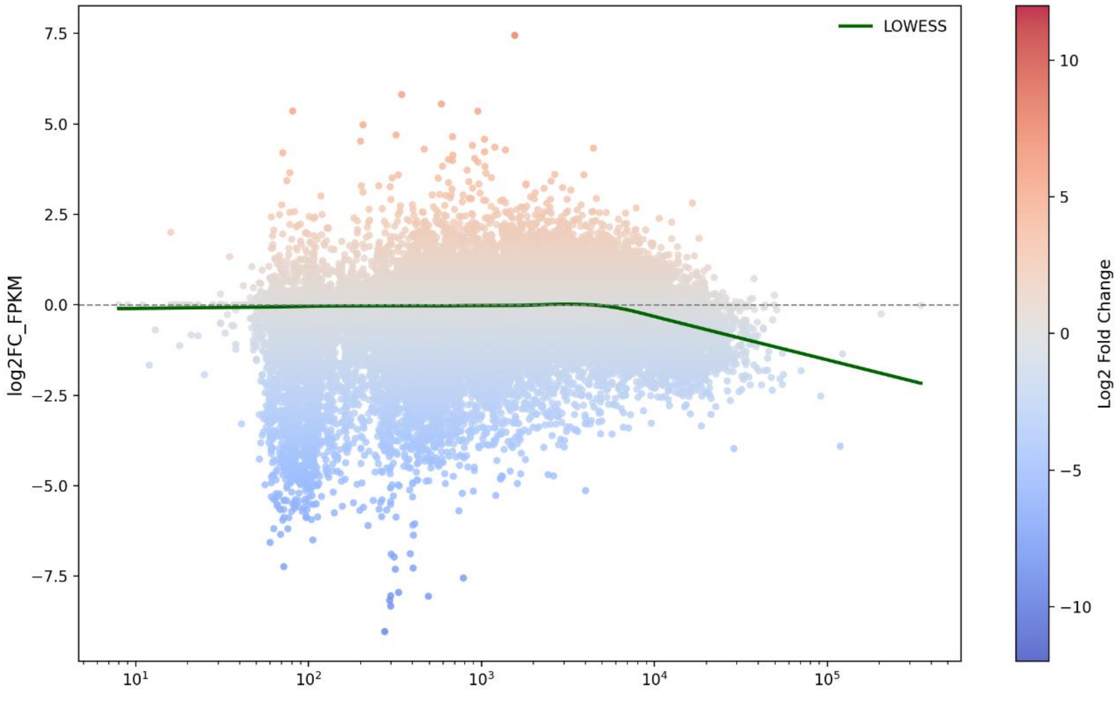

Supplement: Supplementary file 7 — Supplementary Material 7. [file 12864_2026_12944_MOESM7_ESM.pdf]
