# Supplementary material for: Poly(A)+ selection limits detection of long and alternatively spliced transcripts compared with rRNA depletion in RNA-Sequencing
Source: BMC Genomics. 2026 May 13;27:591. doi: 10.1186/s12864-026-12944-z (PMC13339413; doi:10.1186/s12864-026-12944-z)
Supplement: Supplementary file 12 — Supplementary Material 12. [file 12864_2026_12944_MOESM12_ESM.docx]

**RNA Seq analysis code**

Workflow


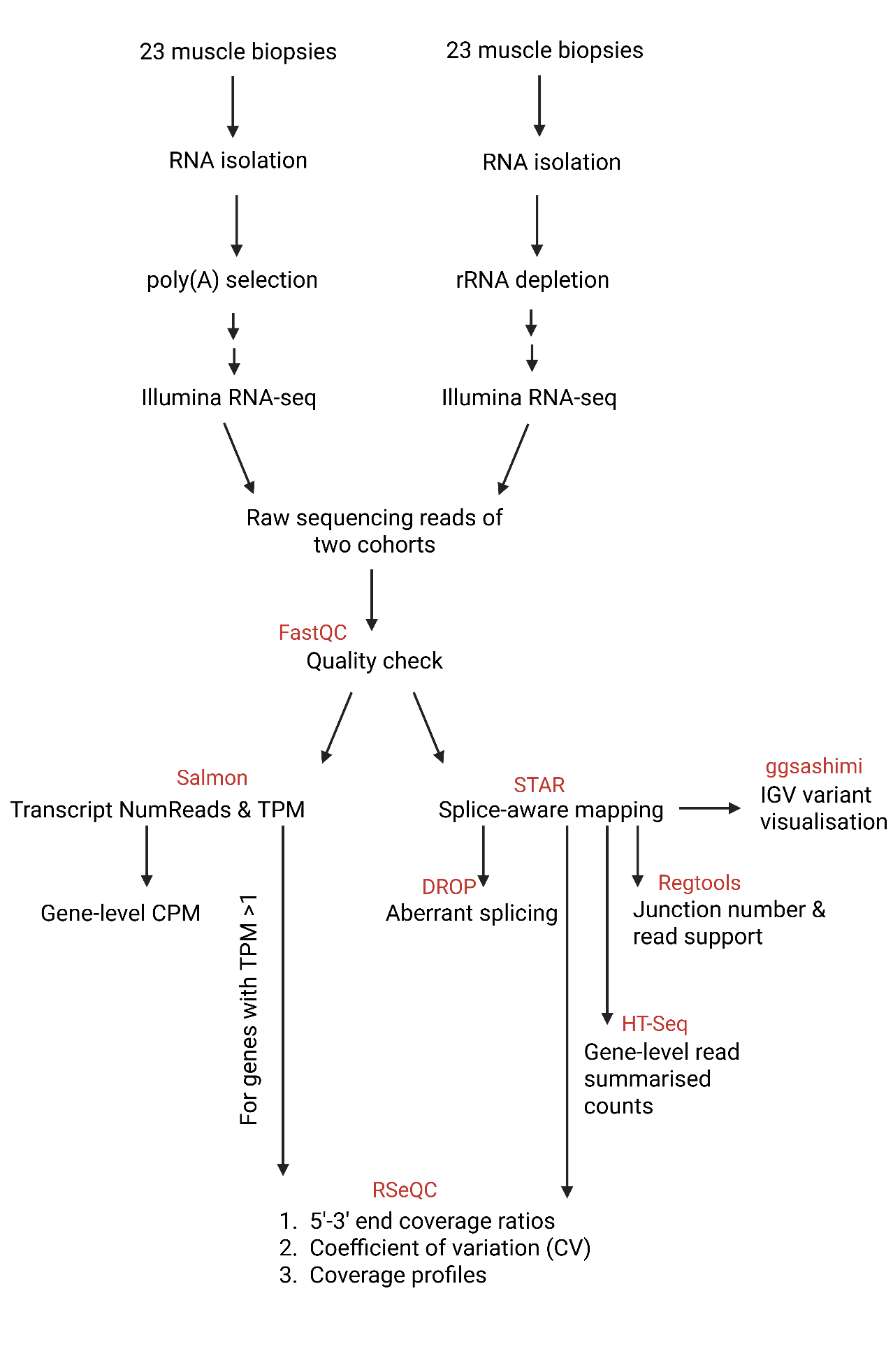


1. **FastQC**

${runFastQC} -o ${output_directory} *.fq.gz

1. **STAR mapping, bam index and junction annotation**

R1="${fastq_folder}/${SAMPLE}/${SAMPLE}_1.fq.gz" # adjust if your structure differs

R2="${fastq_folder}/${SAMPLE}/${SAMPLE}_2.fq.gz"

# ---- 1) STAR alignment (one sample) ----

${runStar} --genomeDir "${genomeDir}/" \

--readFilesIn "${R1}" "${R2}" \

--readFilesCommand zcat \

--genomeLoad NoSharedMemory \

--outFileNamePrefix "${OUTDIR}/${SAMPLE}" \

--outFilterType BySJout \

--outFilterMultimapNmax 20 \

--alignSJoverhangMin 8 \

--alignSJDBoverhangMin 1 \

--outFilterMismatchNmax 999 \

--outFilterMismatchNoverReadLmax 0.04 \

--alignIntronMin 20 \

--alignIntronMax 1000000 \

--alignMatesGapMax 1000000 \

--outSAMunmapped Within \

--outSAMattributes All \

--outSAMtype BAM SortedByCoordinate \

--outSAMheaderHD @HD VN:1.6 SO:coordinate \

--sjdbScore 1 --outMultimapperOrder Random \

--limitOutSJcollapsed 2000000 \

--limitBAMsortRAM 16106127360 \

--twopassMode Basic \

--runThreadN 6

BAM="${OUTDIR}/${SAMPLE}Aligned.sortedByCoord.out.bam"

# ---- 2) BAM index) ----

/usr/bin/samtools index "${BAM}"

# ---- 3) Junction extraction----

${regtools} junctions extract -s 1 -o "${OUTDIR}/${SAMPLE}_junction.bed" "${BAM}"

# ---- 4) Junction annotation----

${regtools} junctions annotate \

-o "${OUTDIR}/${SAMPLE}_junction_annotated.bed" \

"${OUTDIR}/${SAMPLE}_junction.bed" "${REF}" "${GTF}"

1. **From junction.bed file : total junctions, total reads, mean reads per junction**

#!/usr/bin/env python3

import argparse

import pandas as pd

def main():

ap = argparse.ArgumentParser()

ap.add_argument("--gene_bed12", required=True)

ap.add_argument("--junction_bed12", required=True)

ap.add_argument("--sample", required=True)

ap.add_argument("--group", required=True) # polyA or riboD

ap.add_argument("--out_tsv", required=True)

a = ap.parse_args()

# gene BED12: chrom start end name score strand ...

gcols = ["chrom","start","end","gene","score","strand",

"thickStart","thickEnd","itemRgb","blockCount","blockSizes","blockStarts"]

genes = pd.read_csv(a.gene_bed12, sep="\t", header=None, names=gcols, dtype=str)

genes["start"] = pd.to_numeric(genes["start"], errors="coerce")

genes["end"] = pd.to_numeric(genes["end"], errors="coerce")

genes = genes.dropna(subset=["chrom","start","end","gene"]).copy()

genes["gene_length_bp"] = (genes["end"] - genes["start"]).astype("Int64")

# junction BED12 (regtools): score = supporting reads

jcols = ["chrom","start","end","junc_id","score","strand",

"thickStart","thickEnd","itemRgb","blockCount","blockSizes","blockStarts"]

juncs = pd.read_csv(a.junction_bed12, sep="\t", header=None, names=jcols, dtype=str)

juncs["start"] = pd.to_numeric(juncs["start"], errors="coerce")

juncs["end"] = pd.to_numeric(juncs["end"], errors="coerce")

juncs["score"] = pd.to_numeric(juncs["score"], errors="coerce").fillna(0.0)

juncs = juncs.dropna(subset=["chrom","start","end"]).copy()

# prep output (one row per gene)

out = genes[["gene","chrom","start","end","strand","gene_length_bp"]].copy()

out.insert(0, "group", a.group)

out.insert(0, "sample", a.sample)

out["n_junctions"] = 0

out["total_junction_reads"] = 0.0

gene_to_i = {g:i for i,g in enumerate(out["gene"].astype(str).tolist())}

# per-chrom scan

genes = genes.sort_values(["chrom","start","end"]).reset_index(drop=True)

juncs = juncs.sort_values(["chrom","start","end"]).reset_index(drop=True)

for chrom, gdf in genes.groupby("chrom", sort=False):

jdf = juncs[juncs["chrom"] == chrom]

if jdf.empty:

continue

gdf = gdf.sort_values("start").reset_index(drop=True)

gs = gdf["start"].astype(int).tolist()

ge = gdf["end"].astype(int).tolist()

gn = gdf["gene"].astype(str).tolist()

gi, n = 0, len(gs)

for _, jr in jdf.iterrows():

js, je, sc = int(jr["start"]), int(jr["end"]), float(jr["score"])

while gi < n and ge[gi] <= js:

gi += 1

gj = gi

while gj < n and gs[gj] < je:

if (je > gs[gj]) and (js < ge[gj]): # overlap

oi = gene_to_i.get(gn[gj])

if oi is not None:

out.at[oi, "n_junctions"] += 1

out.at[oi, "total_junction_reads"] += sc

gj += 1

out["mean_reads_per_junction"] = out["total_junction_reads"] / out["n_junctions"].replace(0, pd.NA)

out = out[["sample","group","gene","chrom","start","end","strand","gene_length_bp",

"n_junctions","total_junction_reads","mean_reads_per_junction"]]

out.to_csv(a.out_tsv, sep="\t", index=False)

if __name__ == "__main__":

main()

1. **IGV pics from .bam ggsashimi**

ggsashimi.py [-h] -b BAM -c COORDINATES [-o OUT_PREFIX] [-S OUT_STRAND]
                    [-M MIN_COVERAGE] [-j JUNCTIONS_BED] [-g GTF] [-s STRAND]
                    [--shrink] [-O OVERLAY] [-A AGGR] [-C COLOR_FACTOR]
                    [--alpha ALPHA] [-P PALETTE] [-L LABELS] [--fix-y-scale]
                    [--height HEIGHT] [--ann-height ANN_HEIGHT]
                    [--width WIDTH] [--base-size BASE_SIZE] [-F OUT_FORMAT]
                    [-R OUT_RESOLUTION] [--debug-info] [--version]

1. **TPM with Salmon**

salmon quant -i gencode.v39.transcripts_index -l A \

-1 "${folder}/${samp}_1.fq.gz" \

-2 "${folder}/${samp}_2.fq.gz" \

-p 8 --validateMappings -o "transcript_counts/${samp}_quant"

1. **CPM gene wise calculation from Salmon**

import pandas as pd

df = pd.read_csv("quant.sf", sep="\t")

split = df["Name"].str.split("|", expand=True)

df["GeneID"] = split[1]

df["GeneName"] = split[4]

gene_counts = df.groupby(["GeneID","GeneName"])["NumReads"].sum()

cpm = gene_counts / gene_counts.sum() * 1e6

cpm.to_csv("gene_CPM.tsv", sep="\t", header=["CPM"])

1. **HT-Seq read summarization, FPKM, CPM**

${runPython} -m HTSeq.scripts.count --format=bam --order=pos --stranded=reverse --additional-attr=gene_name SAMPLE.bam "${gtf_file}" > "${output_folder}/SAMPLE_counts.txt"

df = pd.read_csv("counts.csv")

sample_cols = df.columns.difference(["GeneID","GeneName","gene_length_bp"])

counts = df[sample_cols].astype(float)

cpm = counts.div(counts.sum()) * 1e6

fpkm = counts.div(df["gene_length_bp"]/1e3, axis=0) \

.div(counts.sum()/1e6)

1. **Gene Body coverage, CV and end bias calculation**

**For all genes in reference.bed12 file:**

geneBody_coverage.py -r reference.bed12 -i SAMPLE.bam -o SAMPLE_geneBody

**For each gene in reference.bed12 file:**

BED="GENE.bed12"

BAM="SAMPLE.bam"

OUT="GENE_SAMPLE"

nohup geneBody_coverage.py -r "$BED" -i "$BAM" -o "$OUT" > "$OUT.log" 2>&1 &

**CV and coverage end bias:**

import pandas as pd

import numpy as np

df = pd.read_csv("SAMPLE.geneBodyCoverage.txt", sep="\t", index_col=0)

sample = df.index[0] # first (or only) sample

values = pd.to_numeric(df.loc[sample], errors="coerce").dropna()

cv = np.std(values) / np.mean(values) if len(values) and np.mean(values) != 0 else np.nan

five_prime = values.iloc[:20].mean()

three_prime = values.iloc[-20:].mean()

ratio = five_prime / three_prime if three_prime != 0 else np.nan

result = pd.DataFrame([{

"Sample": sample,

"CV": cv,

"FiveToThree_Ratio": ratio

}])

result.to_csv("geneBody_summary.csv", index=False)

1. **CV vs length bins plot**

import pandas as pd

import numpy as np

import matplotlib.pyplot as plt

# ---- ONE INPUT FILE ----

df = pd.read_csv("input.csv") # must have: Length (bp) + CV

# ---- transform ----

df["gene_length_kb"] = df["Length"] / 1000

df = df[df["gene_length_kb"] >= 1].copy()

df["log2_CV"] = np.log2(df["CV"] + 1e-6)

# ---- pick ONE bin scheme (edit) ----

bins = [1, 5, 10, 15, 25, 40, 50]

labels = ["1-5kb","5-10kb","10-15kb","15-25kb","25-40kb","40-50kb"]

df["length_bin"] = pd.cut(df["gene_length_kb"], bins=bins, labels=labels, include_lowest=True)

# ---- filter ----

df = df[(df["CV"] <= 2.0) & df["length_bin"].notna()].copy()

# ---- optional quick plot ----

df.boxplot(column="log2_CV", by="length_bin", grid=False)

plt.suptitle("")

plt.title("log2(CV) by length bin")

plt.xlabel("Length bin (kb)")

plt.ylabel("log2(CV)")

plt.xticks(rotation=45)

plt.tight_layout()

plt.savefig("log2CV_boxplot.png", dpi=300)

plt.close()

# ---- save per-gene table ----

df.to_csv("per_gene_log2CV_binned.tsv", sep="\t", index=False)

1. **5’-3’ end comparison of specific genes**

import pandas as pd, numpy as np

import matplotlib.pyplot as plt, seaborn as sns

from scipy.stats import mannwhitneyu

df = pd.read_csv("input.csv")

df["log2_5to3"] = np.log2(df["FiveToThree_Ratio"] + 1e-6)

gene = "TTN"

sub = df[df["GeneName"] == gene].copy()

poly = sub[sub["Protocol"]=="poly(A)+"]["log2_5to3"]

ribo = sub[sub["Protocol"]=="RiboD"]["log2_5to3"]

p = mannwhitneyu(poly, ribo, alternative="two-sided").pvalue if len(poly)>1 and len(ribo)>1 else np.nan

sns.boxplot(data=sub, x="Protocol", y="log2_5to3", showfliers=False)

sns.stripplot(data=sub, x="Protocol", y="log2_5to3", jitter=0.2, alpha=0.8)

plt.title(f"{gene} (p={p:.2e})" if np.isfinite(p) else gene)

plt.ylabel("log2(5′/3′ coverage ratio)")

plt.tight_layout()

plt.savefig("gene_5to3_bias.png", dpi=300)

plt.show()

1. **Coverage plot for all samples per gene**

import pandas as pd, matplotlib.pyplot as plt

genes = ["TTN","OBSCN","MYOD1"]

pct = [10,20,30,40,50,60,70,80,90,100]

def gtext(p):

d = pd.read_csv(p, sep="\t", index_col=0)

d.columns = pd.to_numeric(d.columns, errors="coerce")

d = d.apply(pd.to_numeric, errors="coerce")

return d.div(d.sum(1), 0)

fig, ax = plt.subplots(2, len(genes), figsize=(5*len(genes), 8), squeeze=False)

for j,g in enumerate(genes):

A, R = gtext(f"{g}_combined_raw_polyA.gtext"), gtext(f"{g}_combined_raw_riboD.gtext")

xA = [p for p in pct if p in A.columns]; xR = [p for p in pct if p in R.columns]

for _,r in A[xA].iterrows(): ax[0,j].plot(xA, r.values, lw=2.5, alpha=.8, color="#66c2a5")

for _,r in R[xR].iterrows(): ax[1,j].plot(xR, r.values, lw=2.5, alpha=.8, color="#fc8d62")

ax[0,j].set_title(f"{g} – poly(A)+"); ax[1,j].set_title(f"{g} – RiboD")

ax[0,j].set_xlabel("Percentile"); ax[1,j].set_xlabel("Percentile")

ax[0,j].set_ylabel("RowSum norm"); ax[1,j].set_ylabel("RowSum norm")

fig.tight_layout()

fig.savefig("all_genes_samples_rowsum_polyA_vs_riboD_muscle.png", dpi=800)

plt.show()

1. **ReSEQC junction annotation and saturation**

REF_BED="gencode.v39.combined.bed12"

cd "$work_folder"

bam="SAMPLE.bam"

outdir="OUTPUT_DIR"

sample=$(basename "$bam" .bam)

mkdir -p "$outdir"

junction_annotation.py -i "$bam" -r "$REF_BED" -o "$outdir/${sample}_junction_annotation"

junction_saturation.py -i "$bam" -r "$REF_BED" -o "$outdir/${sample}_junction_saturation"

1. **Gene biotype**

import pandas as pd, numpy as np, re

CPM_THR, MIN_REPS = 1.0, 2

bt = pd.read_excel("gene_biotypes.xlsx")

bt["ENSG"] = bt.filter(like="EnsemblGeneID").iloc[:,0].astype(str).str.extract(r"^(ENSG\d+)")[0]

m_ensg = bt.dropna(subset=["ENSG"]).drop_duplicates("ENSG").set_index("ENSG")["Biotype"]

m_name = bt.drop_duplicates("GeneName").set_index("GeneName")["Biotype"]

df = pd.read_csv("gene_CPM.tsv", sep="\t")

sample_cols = [c for c in df.columns if c not in ["GeneID","GeneName","GeneID_clean","EnsemblGeneID_clean","GeneName_clean"]]

keep = (df[sample_cols].apply(pd.to_numeric, errors="coerce").fillna(0.0) >= CPM_THR).sum(1) >= MIN_REPS

ensg = df.loc[keep, "GeneID"].astype(str).str.extract(r"^(ENSG\d+)")[0]

gname = df.loc[keep, "GeneName"].astype(str).str.strip()

bio = ensg.map(m_ensg).fillna(gname.map(m_name)).fillna("others")

def collapse(s):

s = str(s)

return "protein_coding" if s=="protein_coding" else ("lncRNA" if "lnc" in s.lower() else ("miRNA" if s.lower()=="mirna" else ("snRNA" if s.lower()=="snrna" else "others")))

counts = bio.map(collapse).value_counts().reindex(["protein_coding","lncRNA","miRNA","snRNA","others"], fill_value=0)

out = pd.DataFrame({"category": counts.index, "n_genes": counts.values, "pct": counts.values / counts.sum() * 100})

out.to_csv("biotype_summary.tsv", sep="\t", index=False)

1. **Read distribution**

REF_BED="gencode.v39.combined.sorted.bed"

bam="SAMPLE.bam"

out_dir="OUTPUT_DIR"

sample=$(basename "$bam" .bam)

mkdir -p "$out_dir"

read_distribution.py -i "$bam" -r "$REF_BED" > "$out_dir/${sample}_read_distribution.txt"

1. **CPM expression estimates**

import numpy as np

import pandas as pd

import matplotlib.pyplot as plt

from statsmodels.nonparametric.smoothers_lowess import lowess

# ---- LOAD ----

polyA = pd.read_csv("gene_polyA_CPM.tsv", sep="\t", index_col=[0,1])

ribo = pd.read_csv("gene_riboD_CPM.tsv", sep="\t", index_col=[0,1])

gl = pd.read_csv("gene_length_ordered.csv").rename(columns={"gene_id":"GeneID"})

# ---- MEAN + LOG2FC ----

df = pd.DataFrame({

"GeneID": [i[0].split(".")[0] for i in polyA.index],

"PolyA_mean": polyA.mean(1).values,

"RiboD_mean": ribo.reindex(polyA.index).mean(1).values

})

df["Log2FC"] = np.log2((df["PolyA_mean"] + 1) / (df["RiboD_mean"] + 1))

gl["GeneID"] = gl["GeneID"].astype(str).str.split(".").str[0]

df = df.merge(gl[["GeneID","Length"]], on="GeneID", how="inner").dropna()

df.to_csv("scatter_input.tsv", sep="\t", index=False)

# ---- LOWESS ----

df["log10_Length"] = np.log10(df["Length"].astype(float))

lw = lowess(df["Log2FC"], df["log10_Length"], frac=0.1)

# ---- PLOT ----

plt.style.use('seaborn-v0_8-darkgrid')

plt.figure(figsize=(12,8))

sc = plt.scatter(

df["Length"], df["Log2FC"],

c=df["Log2FC"], cmap="coolwarm",

alpha=0.6, s=50

)

plt.plot(10**lw[:,0], lw[:,1], color="darkgreen", linewidth=1.5, label="LOWESS")

plt.axhline(0, color="gray", linestyle="--", linewidth=1)

plt.xscale("log")

plt.xlabel("Gene Length (bp, log scale)")

plt.ylabel("Log2FC (PolyA / RiboD)")

plt.title("Gene Length vs Log2 Fold Change (with LOWESS)")

plt.colorbar(sc, label="Log2 Fold Change (Log2FC)")

plt.legend()

plt.tight_layout()

plt.show()

1. **JBrowse2 analysis**

**STEP 1 Generate Normalized Coverage (BigWig)**

samtools faidx genome.fa

cut -f1,2 genome.fa.fai > genome.chrom.sizes

1B. Generate normalized bedGraph (CPM normalization)

samtools view -c -F 260 polyA.bam

samtools view -c -F 260 riboD.bam

Scale factor = 1,000,000 / 50,000,000 = 0.02

bedtools genomecov -ibam polyA.bam -bg -scale 0.02 > polyA_CPM.bedGraph

1C. Convert to BigWig

wget http://hgdownload.soe.ucsc.edu/admin/exe/linux.x86_64/bedGraphToBigWig

chmod +x bedGraphToBigWig

./bedGraphToBigWig polyA_CPM.bedGraph genome.chrom.sizes polyA_CPM.bw

./bedGraphToBigWig riboD_CPM.bedGraph genome.chrom.sizes riboD_CPM.bw

**STEP 2 Create Splice Junction Arcs from STAR SJ.out.tab**

awk 'BEGIN{OFS="\t"}

{

start=$2-1;

end=$3;

score=$7;

print $1,start,end,"JUNC_"NR,score,$4,start,end,"0,0,255",2,"1,1","0,"(end-start)

}' SJ.out.tab > junctions.bed

**STEP 3 Download JBrowse2 and load files**

python3 -m http.server 8080

Open the URL in browser and load tracks

1. **Infer Sex from RNA-Seq data**

library(rnatoolbox)

polyA <- scan("polyA_samples.txt", what="character", sep="\n")

riboD <- scan("riboD_samples.txt", what="character", sep="\n")

bamVec <- unique(c(polyA, riboD))

names(bamVec) <- gsub("\\..*", "", basename(bamVec))

ratioCuftoff=0.05

chrRatio <- classifySex(

bamFiles = bamVec,

plotFile = "classifySex_groups.png",

height = 600, width = 1100, fileFormat = "png", pointsize = 14,

numChr = "chrY", denumChr = "chrX",

mar = c(24.1, 4.1, 1.1, 1.1),

hLine = 0.05, main = "")
